# AI Chatbots in Pediatric Orthopedics: How Accurate Are Their Answers to Parents’ Questions on Bowlegs and Knock Knees?

**DOI:** 10.3390/healthcare13111271

**Published:** 2025-05-27

**Authors:** Ahmed Hassan Kamal

**Affiliations:** Division of Orthopedics, Department of Surgery, College of Medicine, King Faisal University, Al-Ahsa 31982, Saudi Arabia; aeltair@kfu.edu.sa

**Keywords:** AI chatbots, knee deformities, parental concerns, health information accuracy

## Abstract

Background/Objectives: Large-language modules facilitate accessing health information instantaneously. However, they do not provide the same level of accuracy or detail. In pediatric orthopedics, where parents have urgent concerns regarding knee deformities (bowlegs and knock knees), the accuracy and dependability of these chatbots can affect parent decisions to seek treatment. The goal of this study was to analyze how AI chatbots addressed parental concerns regarding pediatric knee deformities. Methods: A set of twenty standardized questions, consisting of ten questions each on bowlegs and knock knees, were designed through literature reviews and through analysis of parental discussion forums and expert consultations. Each of the three chatbots (ChatGPT, Gemini, and Copilot) was asked the same set of questions. Five pediatric orthopedic surgeons were then asked to rate each response for accuracy, clarity, and comprehensiveness, along with the degree of misleading information provided, on a scale of 1–5. The reliability among raters was calculated using intraclass correlation coefficients (ICCs), while differences among the chatbots were assessed using a Kruskal–Wallis test with post hoc pairwise comparisons. Results: All three chatbots displayed a moderate-to-good score for inter-rater reliability. ChatGPT and Gemini’s scores were higher for accuracy and comprehensiveness than Copilot’s (*p* < 0.05). However, no notable differences were found in clarity or in the likelihood of giving incorrect answers. Overall, more detailed and precise responses were given by ChatGPT and Gemini, while, with regard to clarity, Copilot performed comparably but was less thorough. Conclusions: There were notable discrepancies in performance across the AI chatbots in providing pediatric orthopedic information, which demonstrates indications of evolving potential. In comparison to Copilot, ChatGPT and Gemini were relatively more accurate and comprehensive. These results highlight the persistent requirement for real-time supervision and stringent validation when employing chatbots in the context of pediatric healthcare.

## 1. Introduction

The use of artificial intelligence (AI) technologies has transformed the distribution and retrieval processes of medical information [1]. One of the most notable innovations stemming from technological advancement is that of chatbots (large-language modules, i.e., LLMs), which are automated agents developed to facilitate the delivery of accurate, timely, and user-friendly health information [2]. These digital interfaces are becoming more popular in an age when parents addressing pediatric healthcare issues are only a few keystrokes away from obtaining the needed information [3]. Nevertheless, the speed at which chatbots can offer information stands in stark contrast to the need to ensure that this information is accurate, particularly for time-sensitive conditions [4].

Conditions like genu varum (bowlegs) and genu valgum (knock knees) represent concerns that impact the child’s physical appearance and their future musculoskeletal well-being [5]. Timely intervention at the right stage and management of these conditions is important to avoid complications and nurture healthy physical growth [6]. The child’s guardian, more often than not, is the first to notice unusual patterns or misalignments of the child’s limbs in relation to gait cycle and seeks guidance from various platforms, including chatbots [3]. Considering how multifaceted and detailed pediatric orthopedic issues are, the accuracy of a chatbot’s response is critical in determining whether a child’s orthopedic assessment is postponed or preemptively altered, potentially leading to misguided attempts at self-treatment [7].

Prior research has looked into the effectiveness and functionality of chatbots across various branches of healthcare. The works on the use of chatbots in symptom checking and in mental health services tend to showcase the advantages and imperfections of such technologies [8]. For example, some studies have shown that while chatbots may efficiently dispense basic general health information and conduct preliminary evaluations, they often lack the ability to provide the detailed clinical context that is important for diagnostic and management decision-making processes [2,9]. In children’s healthcare, some preliminary studies have indicated that chatbot answers often fail to meet the specificity needed to address the knee deformity issues that some parents may be facing [7]. These findings highlight the importance of robust comparative assessments involving pediatric orthopedic guidelines and expert opinions on consultant technologies in orthopedics for children [6].

To the best of our knowledge, there is limited literature regarding the validity of LLMs in addressing parental queries about pediatric knee deformities. The goals of this study were as follows:

- Assessing the accuracy of chatbot responses to parental questions concerning pediatric knee deformities.
- Offering an open, replicable evaluation framework and benchmarking dataset for future work on health-oriented chatbots.
- Evaluating the responses against the clinical standards and expertise of pediatric orthopedic practitioners to assess their reliability. Other aspects of the evaluation include the clinical accuracy, clarity, and a completeness devoid of ambiguity, which may indicate a lack of precision that could postpone essential medical action.

## 2. Materials and Methods

### 2.1. Study Design

This is an analytical cross-sectional study intended to evaluate how effectively chatbots interact compared to established clinical guidelines and expert views in pediatric orthopedic surgery. This study aims to assess the consistency and common sense in the answers given by chatbots to some basic queries on bowlegs and knock knees.

### 2.2. Data Collection

#### 2.2.1. Chatbot Selection

This study incorporated a sample of general purpose chatbots that have gained considerable traction within the general population. These chatbots were selected according to the following principles:

- The most frequently used chatbot by the Saudi population according to the report from the Saudi Center for Public Opinion Polling (SCOP) [10].
- Popularity and user ratings in app stores or online platforms.
- Timely access and availability during the period of the study.

The included chatbots were ChatGPT version 4.0, Google Gemini 2.0, and Microsoft Copilot.

#### 2.2.2. Question Formulation

Using a literature review, parental forums, and collection of real-world queries from the FAQ sections of ten evidence-based patient information websites (Ortho Info-AAOS, POSNA Ortho Kids, Nemours Kids Health, HealthyChildren.org, Boston Children’s Hospital, Seattle Children’s Hospital, Royal Children’s Hospital Melbourne, and NHS Inform [11,12,13,14,15,16,17,18,19,20,21]), questions specifically related to children’s genu varum and genu valgum were gathered (which amounted to 30 candidate questions). Two pediatric orthopedic surgeons cross-checked these items against queries raised during clinic visits and removed six that involved scenarios unencountered in practice, like stem-cell injections. Items with identical meanings were merged, lowering the total to 23 unique questions. The expert panel included five senior pediatric orthopedic surgeons from the five local hospitals in the authors’ area who kindly consented to review the questions and subsequently rate the responses, and they evaluated all of the questions on two aspects: their phrasing and clarity, alongside a focus on parental-influenced decision making. Cost-related queries of specific regions along with adult or experimental topics (3D printing and knee replacement) were eliminated. The final number of questions was 20 questions (10 about bowlegs and 10 about knock knees). The questions included the following elements (Supplementary Materials File S1):

- Description of the condition.
- Normal developmental variations.
- Etiological factors and risk factors.
- Diagnostic procedures and tests used.
- Treatment and its effectiveness.
- Complications.
- Timing of medical intervention.

#### 2.2.3. Creating Responses

To achieve uniformity, all of the chatbots were asked the same questions at once. Responses were recorded accurately and classified for further examination. The first response produced by the chatbot was logged and utilized for the evaluation.

### 2.3. Evaluating Responses

#### 2.3.1. Appraisal of the Accuracy

Responses were assessed by a panel of five practicing pediatric orthopedic surgeons with a minimum experience of five years who had a consensus on the following four parameters of the appraisal (and they rated each answer independently based on these parameters).

- Accuracy: Ensuring accuracy is especially vital for pediatric orthopedic conditions due to its prominence in early intervention as a specialist discipline. Inaccurate technology-derived advice risks delaying care through mismanaged awareness of the proper steps to take and, in some cases, due to overly complicating intervention strategies that could otherwise be straightforward [2,22].
- Clarity: Focusing on the reception of the chatbot’s answer by parents, many of whom do not possess medical training, pertains to clarity. Reducing anxiety or confusion toward a child’s condition relies on the ability to foster appropriate information control [23,24].
- Comprehensiveness: Looking into answering the question thoroughly by providing its components, such as the causatives, their treatment, and the timing of care seeking, define comprehensiveness. Failure to answer comprehensively risks providing incomplete responses that the user deems crucial. This reduces the educational value of chatbot interactions [2,24].
- Risk of Misleading Information: The risk of accurate-sounding yet false information is increasingly problematic with the advent of AI models. The possible hazards of conversational agents providing contextualized and non-contextualized dangerous advice have been documented in multiple studies [22,24,25].

An average for each response was calculated from the overall scores given for each criterion. Each answer was rated on a 5-point scale (1 = unsatisfactory to 5 = exceptional) in all four set criteria.

#### 2.3.2. Readability Assessments Using a Flesch–Kincaid Readability Test

The Flesch–Kincaid readability test measures the difficulty of a text by assessing its sentence length and the syllable count per word. Textual complexity was measured with the Flesch–Kincaid Grade-Level (FKGL) index, which assesses the reading level of a text by estimating the school grade that is associated with understanding it, and it is based on average sentence length and average syllables per word using the following formula: FKGL = 0.39 × (words ÷ sentences) + 11.8 × (syllables ÷ words) − 15.59 [26,27]. The index was initially created for the U.S. Navy, and its predictive validity as estimating comprehension has been reliably documented, which is why it is commonplace in the evaluation of educational materials, including patient instructions, the health information available online, and documents requiring informed consent from patients in various fields of medicine [28,29]. As for every answer provided by the chatbot, the text was copied in full to the Hemingway Editor online calculator [30] and the online Character calculator [31], where grade-level estimation was performed based on the original formula with no prior modifications to the text.

### 2.4. Statistical Analysis

The data were analyzed using IBM SPSS version 29 (IBM Corp., Armonk, NY, USA) and JASP software (Version 0.19.3). The median and range were reported for each chatbot to summarize the performance. Evaluation of the inter-rater reliability was performed using the intraclass correlation coefficient (ICC) and its 95% confidence interval as these statistic capture the absolute agreement of multiple raters on the interval or ordinal scales that fit our five-point expert ratings. In addition, more than two reviewer’s evaluations, using a two-way mixed-effects model with an absolute agreement definition of ICC, was also utilized. The average rating for each domain was used in a Kruskal–Wallis test followed by a Dunn post hoc test to assess the differences in performance across chatbots because the rank score distributions were not normally distributed. The effect sizes were measured by rank-biserial correlation, which yielded ranked biserial (r). A *p* value of less than 0.05 for the 95% confidence interval was considered significant.

### 2.5. Ethical Considerations

The use of content produced by a chatbot means that there are no participants involved in the research; thus, formal ethical approval may not be necessary. Even so, the precision and possible consequences of errors in the information provided had to be assessed carefully in order to mitigate the potential harms of exposing misleading information.

## 3. Results

### 3.1. Inter-Rater Reliability

Inter-observer agreement was assessed for both the bowleg and knock knees question sets using a two-way mixed-effects model (with an absolute agreement definition of the intraclass correlation coefficient (ICC)). Then, the average-measures ICC and its 95% confidence interval were focused upon (i.e., a ICC value below 0.5 indicates poor reliability, a value between 0.5 and 0.75 indicates moderate reliability, and a value between 0.75 and 0.9 indicates good reliability).

#### 3.1.1. Bowlegs Responses

The bowleg analyses indicated overall moderate-to-good reliability. Specifically, for “Accuracy”, the ChatGPT ratings had an ICC of 0.750 (95% CI: 0.268–0.932, *p* = 0.006), Gemini reached 0.825 (95% CI 0.487–0.953, *p* < 0.001), and Copilot delivered 0.745 (95% CI 0.253–0.931, *p* = 0.007).

For “Clarity”, ChatGPT demonstrated an ICC of 0.828 (95% CI 0.496–0.954, *p* < 0.001), Gemini attained 0.819 (95% CI 0.471–0.951, *p* = 0.001), and Copilot produced 0.641 (95% CI: −0.050 to 0.903, *p* = 0.031).

In terms of “Comprehensiveness”, ChatGPT reached 0.812 (0.449–0.949, *p* = 0.001), Gemini achieved 0.750 (95% CI 0.268–0.932, *p* = 0.006), and Copilot delivered a moderate score at 0.659 (95% CI 0.000–0.908, *p* = 0.025).

Finally, for “Risk of Misleading Information”, ChatGPT, Gemini, and Copilot all delivered ICCs of 0.644, 0.605, and 0.594, respectively—each indicating a moderate reliability.

#### 3.1.2. Knock Knees Responses

For knock knees, the results also varied from moderate to good. Under “Accuracy”, ChatGPT delivered a moderate score at an ICC of 0.594 (95% CI −0.189 to 0.890, *p* = 0.049), whereas Gemini attained good reliability (ICC = 0.793, 0.395–0.944, *p* = 0.002) and Copilot delivered a moderate one at 0.605 (95% CI −0.158 to 0.893, *p* = 0.045).

In the “Clarity” domain, ChatGPT reached an ICC = 0.745 (95% CI 0.253–0.931, *p* = 0.007), Gemini had an ICC = 0.646 (95% CI −0.037 to 0.904, *p* = 0.029), and Copilot similarly showed an ICC = 0.641 (95% CI −0.050 to 0.903, *p* = 0.031), marking the strongest agreement.

For “Comprehensiveness,” ChatGPT and Gemini showed ICCs around 0.67, whereas Copilot had an ICC = 0.618 (95% CI −0.118 to 0.897, *p* = 0.039).

Finally, regarding “Risk of Misleading Information” ChatGPT, Gemini, and Copilot each showed moderate reliability (all ICCs ~0.60–0.64), with negative lower bounds suggesting notable variability.

In sum, both the bowleg and knock knees sets demonstrated overall moderate-to-good inter-observer agreement. Therefore, the average ratings were utilized for further analysis (Supplementary Materials File S2).

### 3.2. Flesch–Kincaid Readability Test Results

According to the Flesch–Kincaid readability assessments for both “Knock knees” and “Bow legs”, ChatGPT achieved the highest readability for both, scoring 61 for “Knock knees”, which is an 8th-grade level, and 56 for “Bow legs”, which fell into the 10th–12th grades. In contrast, Gemini and Copilot produced more complex text: they both scored 51 for “Knock knees”, which aligns with the 10th–12th grade, while Gemini scored 50 for “Bow legs”, putting it into the 10th–12th grade range. In addition, Copilot scored 48, placing it at college level. These findings indicate that ChatGPT is easier to read while Gemini and Copilot’s content are targeted to more advanced readers (Table 1).

### 3.3. Comparison of Different Chatbots Responses

Across the four dimensions—accuracy, clarity, comprehensiveness, and risk of misleading information—the three models (ChatGPT, Copilot, and Gemini) all exhibited median ratings that were relatively high, i.e., generally above 4.0. For accuracy, ChatGPT and Gemini both had a median of 5.00, suggesting that at least half of the ratings for each were at the maximum value, whereas Copilot’s median was slightly lower (4.67). However, Gemini’s ratings varied more (with a range of 1.67) than those of ChatGPT (with a range of 0.67) and Copilot (with a range of 1.33). In terms of clarity, Copilot (4.83) and Gemini (4.83) shared the highest medians, with ChatGPT just behind at 4.67; ChatGPT’s ratings in clarity also had the largest range (1.67) relative to Copilot and Gemini (1.33 each). For comprehensiveness, both ChatGPT and Gemini again had medians of 5.00, while Copilot’s median was 4.00 with a range of 1.67, indicating more dispersion and lower central tendency on this dimension. Finally, when it came to the risk of wrong answers, Copilot was the only model with a median rating of 5.00, while ChatGPT and Gemini both were at 4.67. Although ChatGPT and Gemini shared the same central tendency here, ChatGPT’s range was 1.33 and Gemini’s was 1.00, showing slightly less spread for Gemini. Overall, these medians and ranges suggest that ChatGPT and Gemini often cluster at the top end of the scale for accuracy and comprehensiveness, with Copilot excelling in clarity and in the perceived likelihood of providing correct answers; however, all three were generally rated favorably, with only minor differences emerging across categories (Table 2).

In the Kruskal–Wallis tests exploring the performance of the three chatbots (Copilot, Gemini, and ChatGPT) and four performance metrics—accuracy, clarity, comprehensiveness, and the capacity to generate wrong answers—the differential performance was established for accuracy (*p* = 0.020) and comprehensiveness (*p* = 0.002), while no differences were noted for clarity (*p* = 0.768) and the likelihood of generating wrong answers (*p* = 0.628) (Table 3). Bonferroni correction pairwise comparisons (Table 4) showed that ChatGPT was significantly more accurate than Copilot (*p* = 0.017), with Copilot showing no significant differences with Gemini. For comprehensiveness, Copilot was significantly lower than both Gemini (*p* = 0.007) and ChatGPT (*p* = 0.009), with both Gemini and ChatGPT scoring equally. In contrast, participants displayed no significant pairwise differences for the other two measures, claiming the chatbots performed similarly. The results support the claim that, while ChatGPT is more accurate than Copilot and both ChatGPT and Gemini outperformed Copilot in comprehensive ness, all three of the chatbots provided comparable performance in clarity and the propensity for being factually inaccurate.

## 4. Discussion

The current research examines how, evaluating the response accuracy against the four dimensions of performance—clarity, thoroughness, understanding, and possibility of inaccuracy—ChatGPT, Gemini, and Copilot respond to common questions posed about pediatric knee deformities. Most cases of pediatric knee alignment problems, particularly genu varum (bow legs) and genu valgum (knock knees), tend to make parents search the internet for information and reassurance [32]. AI-powered chatbots can simplify healthcare communication through the provision of readily accessible explanations. However, notwithstanding that such conditions hold significant value, alongside the complexity associated with differential diagnosis and treatment methodologies, these explanations require dependable, authoritative information [33].

In this study, ChatGPT and Gemini emerged as stronger performers in terms of accuracy and comprehensiveness compared to Copilot, suggesting that they may deliver more detailed and precise explanations about the developmental trajectories of pediatric knee deformities, potential red flags (e.g., significant pain, asymmetric deformities, or functional limitations), and typical treatment pathways, such as observation, bracing, physical therapy, or surgical intervention [10]. These findings support claims made regarding large-language models (LLMs) that undergo training on vast amounts of high-quality data, as such models are better at responding to specific queries that are sophisticated in nature [34,35].

The Kruskal–Wallis tests showed significant differences in how the chatbots performed regarding accuracy (*p* = 0.020) and comprehensiveness (*p* = 0.002), but no differences for clarity (*p* = 0.768) or the chance of providing erroneous answers (*p* = 0.628) were found. In particular, ChatGPT’s accuracy was significantly higher than Copilot’s, as has been observed in prior studies where well-trained models performed superbly in fact-based response tasks. Although Gemini’s accuracy was not statistically different from the other two models, its rank among Copilot and ChatGPT in comprehensive scoring did elevate it beyond the latter’s mimicry. The practical significance is that Gemini and ChatGPT are likely, in agreement with precious study [32], to be more beneficial to parents seeking normal knee alignment explanations because they offer fuller answers on when and how to professionally consult.

The use of chatbots as the first point of contact for information about pediatric knee deformities for caregivers is very important to consider. Providing accurate and detailed answers can greatly reduce unnecessary concern, assist in developmental surveillance, or, if necessary, stimulate prompt medical assessments. Clarity, while relatively consistent across all three of the models, remains very important: parents lacking background knowledge in the medical field tend to be intuitive and interpret complex ideas expressed in straightforward language as simplistic [32]. Nonetheless, the differences that were not statistically significant regarding the likelihood of wrong answers through the chatbots serve as a reminder that all three models, regardless of their straining sophistication, possess a chance of inaccuracy [36].

In pediatric settings, the stakes of inaccurate medical guidance can be high. A chatbot incorrectly dismissing early signs of a pathological condition, such as Blount’s disease, could delay essential interventions that are crucial [37]. Consequently, even high-performing chatbots should be complemented by regular updates to their training data and integrated safety nets—such as disclaimers advising users to confirm critical health information with a qualified healthcare professional.

While all three assessed chatbots possess a conversational interface and a short medico-legal disclaimer, they all differ in ways that may unintentionally mislead users seeking information on limb alignment for their children. Compared to Microsoft’s Copilot, which integrates Bing snippets into its responses, ChatGPT and Google Gemini rely entirely on large-language-model synthesis. Studies by Laymouna et al. and Hassan et al. showed that appended hypertextual citations, even if originating from non-peer-reviewed blogs, lend an aura of validation, amplifying cognitive burden for non-expert users and obscuring a linear course of action [4,37]. None of the systems automatically elicited a child’s sex in this study.

Nadarzynski et al. [38] and Nazi et al. [39] indicate that comparative studies suggest LLMs could provide a better educational service for patients when compared to other online resources, like Google Search and YouTube, because LLMs provide more accurate responses tailored to the educational material’s level of complexity. LLMs offer personalized and interactive guidance, allowing users to engage in dialogues, ask follow-up questions, and receive information in simple terms tailored to their needs. Discussion of YouTube-style patient education videos suggests that their utility into more visually dynamic content does not overcome their relatively lower interactivity or higher variability in quality compared to Google Search, posing increased risks for misinformation due to the open access. The same applies to Google Search; it offers access to numerous pieces of information but fails to provide personalization and interactivity, leading to difficulties identifying the source of quality information. Furthermore, unlike YouTube and Google Search, you do not need to rely on ranking algorithms or content creators for the accessibility of information provided by LLMs when they are tailored to users’ health literacy levels.

Regardless of such benefits, LLMs still suffer from inaccuracies and the so-called “hallucinations”, or the generation of incorrect and misleading information [40]. Notably, healthcare professionals should not regard these systems as replacements for medical consultations or professional guidance. Therefore, it is imperative that both clinicians and patients understand the distinctive limitations and dangers, including the misinformation, inherent to these digital resources for health education.

### Limitations and Future Directions

While the current study draws attention to the varying performance levels of ChatGPT, Gemini, and Copilot, its scope remains constrained by the specific evaluation criteria and limited number of pediatric knee deformity scenarios. Larger-scale studies could incorporate broader clinical case variations. Comparing chatbot-generated advice with that provided by qualified pediatric orthopedic specialists would also help clarify the degree to which these AI systems can approximate professional judgment. Also, more studies should be conducted to assess chatbot responses in local languages.

## 5. Conclusions

In addressing frequently asked questions on pediatric knee deformities, ChatGPT and Gemini demonstrate consistently higher accuracy and comprehensiveness, rendering them potentially more reliable for parents seeking comprehensive background information. Nevertheless, all three of the chatbots share a comparable risk of generating incorrect or incomplete content, underscoring the continued importance of professional oversight, critical appraisal, and up-to-date clinical data sources. These insights inform both the design and the usage of AI chatbots in pediatric medical settings, highlighting the delicate balance between immediate accessibility and the necessity for validated, high-quality information in healthcare communication. These findings enrich the existing evidence base and give developers insights for enhancing AI chatbot performance.

## Figures and Tables

**Table 1:** Results of the Flesch–Kincaid readability tests.

Topic	Chatbot	The Score	The Grade Level
Knock knees responses	ChatGPT	61	8th grade
	Gemini	51	10–12th grade
	Copilot	51	10–12th grade
Bow legs responses	ChatGPT	56	10–12th grade
	Gemini	50	10–12th grade
	Copilot	48	College students

**Table 2:** The average ratings for the responses of the three chatbots.

Dimension	Chatbot	Average	Median	Range
Accuracy	ChatGPT	4.87	5	0.67
	Copilot	4.74	4.67	1.33
	Gemini	4.57	5	1.67
Clarity	ChatGPT	4.68	4.67	1.67
	Copilot	4.70	4.83	1.33
	Gemini	4.70	4.83	1.33
Comprehensiveness	ChatGPT	4.84	5	1
	Copilot	4.83	4	1.67
	Gemini	4.44	5	1.33
Risk of Misleading Information	ChatGPT	4.76	4.67	1.33
	Copilot	4.77	5	1
	Gemini	4.81	4.67	1

**Table 3:** Results of the Kruskal–Wallis tests to detect differences between the chatbots.

Variable	Χ^2^ (df = 2)	*p*-Value
Accuracy	7.810	0.020
Clarity	0.528	0.768
Comprehensiveness	12.021	0.002
Risk of Misleading Information	0.929	0.628

**Table 4:** The results of the Dunn post hoc tests with a Bonferroni correction of the *p* values following the Kruskal–Wallis test.

Variable	Comparison	Test Statistic	*p*-Value	Effect Size (r)
Accuracy	Copilot vs. Gemini	−5.800	0.286	
Accuracy	Copilot vs. ChatGPT	−9.650	0.017	0.507
Accuracy	Gemini vs. ChatGPT	−3.850	0.804	
Clarity	Copilot vs. Gemini	0.95	1	
Clarity	Copilot vs. ChatGPT	2.65	1	
Clarity	Gemini vs. ChatGPT	1.7	1	
Comprehensiveness	Copilot vs. Gemini	−10.950	0.007	0.540
Comprehensiveness	Copilot vs. ChatGPT	−10.650	0.009	0.556
Comprehensiveness	Gemini vs. ChatGPT	0.3	1	
Risk of Misleading Information	Copilot vs. Gemini	3.15	1	
Risk of Misleading Information	Copilot vs. ChatGPT	3	1	
Risk of Misleading Information	Gemini vs. ChatGPT	−0.150	1	

## Data Availability

The datasets used and analyzed during the current study are available from the corresponding author upon reasonable request.

## Supplementary Materials

The following supporting information can be downloaded at: https://www.mdpi.com/article/10.3390/healthcare13111271/s1, File S1: Table (1): shows the question about bowlegs and responses among the three chatbots; File S1 Table (2): shows the question about Knock kneesand responses among the three chatbots; File S2: Table (1) inter rater reliability assessment using intraclass correlation coefficient (ICC) for bowlegs responses; File S2: Table (2) inter rater reliability assessment using intraclass correlation coefficient (ICC) for Knock knees responses.
