# Supplementary material for: AI Chatbots in Pediatric Orthopedics: How Accurate Are Their Answers to Parents’ Questions on Bowlegs and Knock Knees?
Source: Healthcare (Basel). 2025 May 27;13(11):1271. doi: 10.3390/healthcare13111271 (PMC12154324; doi:10.3390/healthcare13111271)
Supplement: Supplementary file 1 [file healthcare-13-01271-s001.zip › Supplementory File S1.pdf]

Table (1) : shows the question about bowlegs and responses among the three chatbots:

	Chatgpt	Gemeni	copilot
Question	Response	Response	
1. What are bowlegs?	**Bowlegs** (medical term: **genu varum**) is a condition where a person's legs curve outward at the knees, causing the feet and ankles to touch while the knees remain apart when standing straight. This creates an arched or "bowed" appearance, similar to the shape of a bow.<br><br>Causes of Bowlegs:<br><br>1. **Physiological:** Common in infants and toddlers due to their fetal position. Typically corrects itself by age 3–4 as they grow.<br>2. **Rickets:** A bone-softening disease caused by a vitamin D deficiency.<br>3. **Blount's Disease:** A growth disorder affecting the shin bone (tibia).<br>4. **Bone fractures or improper healing.**<br>5. **Arthritis:** Especially in older adults, affecting knee joints.<br>6. **Genetics:** Some people naturally have a bow-legged structure.<br><br>Symptoms:<br><br>• Noticeable outward curve of the legs.	Bowlegs, also known as genu varum, is a condition where the legs curve outward at the knees while the feet and ankles touch. This creates a gap between the lower legs, resembling the shape of a bow.<br><br>Here's some key information about bowlegs:<br><br>• **Causes:**<br>o **Physiological bowing:** A common and usually harmless condition in infants and toddlers that typically corrects itself as they grow.<br>o **Blount's disease:** A growth disorder affecting the shinbone	creating a gap between the lower legs and knees when the feet are together. This condition is often noticeable in infants and toddlers as their bones and joints are still developing. In many cases, bowlegs naturally correct themselves as the child grows. However, if the condition persists beyond early childhood or develops in adulthood, it may be due to underlying issues such as:<br><br>• **Rickets:** A deficiency in vitamin D, calcium, or phosphate, leading to softening and weakening of the bones.<br>• **Blount's disease:** A growth disorder affecting the shin bone (tibia).<br>• **Bone infections or fractures:** These can affect the alignment

	• Uneven wear on shoes.<br>• Knee pain in severe cases.<br><br>Treatment:<br><br>• **Observation:** For young children, as it often self-corrects.<br>• **Braces or orthotics:** For moderate cases.<br>• **Surgery:** In severe or persistent cases.	(tibia), causing it to grow unevenly.<br>○ **Rickets:** A bone disease caused by vitamin D deficiency, leading to soft and weakened bones.<br>○ **Skeletal dysplasias:** Genetic conditions affecting bone growth and development.<br>○ **Injury or fracture:** Past injuries or fractures can sometimes lead to bowing.<br>• **Symptoms:**<br>○ A noticeable outward curve of the legs.<br>○ Difficulty walking or running.	and growth of the bones.<br>• **Arthritis:** Joint inflammation can sometimes cause bowlegs in older adults.<br><br>Bowlegs can cause discomfort, difficulty walking, and in severe cases, lead to complications like arthritis or joint problems. Treatment options depend on the underlying cause and the severity of the condition. They may include:<br><br>• **Nutritional supplements:** To address deficiencies.<br>• **Bracing:** To help correct the alignment of the legs in children.<br>• **Physical therapy:** Exercises to strengthen muscles and improve alignment.<br>• **Surgery:** In severe cases, surgery may be necessary to

		- Pain or discomfort in the legs or knees.<br>• **Diagnosis:**<br>- Physical examination.<br>- X-rays to assess the alignment of the bones.<br>• **Treatment:**<br>- **Observation:** Physiological bowing often resolves on its own.<br>- **Bracing:** May be used in young children with Blount's disease.<br>- **Surgery:** May be necessary in severe cases or if other treatments are unsuccessful.<br><br>If you're concerned about bowlegs, it's important to	correct the leg alignment.<br><br>If you have any concerns about bowlegs or think you or someone you know might need treatment, it's best to consult with a healthcare professional.

		consult with a doctor or pediatrician for proper evaluation and guidance. They can determine the underlying cause and recommend the most appropriate treatment options.	
2. When is it normal for a child to have bowlegs?	**Bowlegs are normal in infants and young children due to their fetal position in the womb. Typically, this condition is part of a natural developmental phase. Here’s a breakdown of when it’s normal:**<br><br>**Normal Timeline for Bowlegs in Children:**<br><br>**Infants (0–18 months): Bowlegs are common and normal. Most babies are born with some degree of bowing due to their curled position in the womb.**<br><br>**Toddlers (18–24 months): The bowing might appear more noticeable when they start walking. This is still normal at this stage.**<br><br>**By Age 2–3: The legs usually begin to straighten out naturally.**	It's actually quite common for young children to have bowlegs! Here's a general timeline of when bowlegs are considered normal and when they might be something to keep a closer eye on:<br><br>• **Infancy (Birth to 1 year):** Most babies are born with bowed legs due to their position in the womb. This is perfectly normal.<br>• **Toddlerhood (1 to 3 years):** As toddlers start walking, their legs may straighten out, but some degree of bowing is still very common and usually	joints are still developing, and their legs naturally curve outward. Here’s a general timeline of what to expect:<br><br>• **Newborns to 18 months:** Most babies are born with bowlegs due to their position in the womb. This is perfectly normal and usually not a cause for concern.<br>• **18 months to 3 years:** As children start to walk and bear weight on their legs, their bowlegs may become more noticeable. However, in many cases, this condition begins to

	**By Age 4–5: Most children's legs have straightened significantly or may even show a temporary knock-kneed appearance (knees touch, ankles apart), which is also normal.**<br><br>**By Age 7–8: The legs should reach a more permanent alignment, typically straight.**<br><br>**When to Be Concerned:**<br><br>**Severe bowing: Especially if it worsens after age 2.**<br><br>**Only one leg is bowed.**<br><br>**Associated pain or difficulty walking.**<br><br>**Persists beyond age 3–4 without signs of improvement.**	nothing to worry about.<br>• **Preschool Age (3 to 5 years):** By this age, most children's legs will have straightened significantly. However, mild bowing may still be present and often resolves on its own.<br><br>**When to be more aware:**<br><br>• **Severe bowing:** If the bowing is very noticeable or seems to be getting worse.<br>• **Asymmetrical bowing:** If one leg is more bowed than the other.<br>• **Other symptoms:** If your child has pain, difficulty walking, or any other concerns along with the bowlegs.<br>• **Family history:** If there's a family history of bone	improve around this age.<br>• **3 to 4 years:** By this age, most children's legs have naturally straightened out. If the bowlegs persist beyond this point, it's a good idea to consult with a pediatrician to rule out any underlying conditions.<br><br>Keep in mind that every child develops at their own pace, and some variation is normal. However, if you have concerns about a child's leg alignment, it's always best to seek advice from a healthcare professional to ensure everything is on track.

		diseases or skeletal problems.<br><br>**Important Note:** Even though bowlegs are often normal in young children, it's always a good idea to discuss any concerns you have with your pediatrician. They can assess your child's development and determine if any further evaluation or treatment is needed.<br><br>Sources and related content	
3. What can cause bowlegs in a child?	**Bowlegs in children can be caused by several factors, ranging from normal developmental stages to underlying medical conditions. Here are the main causes:**<br><br>🍼 **1. Physiological Bowlegs (Normal Development):**<br><br>**Common in infants and toddlers due to their fetal position in the womb.**	**1. Physiologic Bowing:**<br><br>• This is the most frequent cause, especially in toddlers. It's basically a normal part of growth and development. Think of it like their legs getting used to being outside the womb and learning to bear weight.	1. **Physiological bowlegs:** Many infants are born with bowlegs due to their position in the womb. This type of bowleggedness is usually normal and typically resolves on its own as the child grows.<br>2. **Rickets:** A condition caused by a deficiency in vitamin

	**Typically corrects itself by age 2–3 without treatment.**<br><br>🦴 **2. Medical Causes of Bowlegs:**<br><br>**Rickets:**<br><br>**A bone-softening condition caused by vitamin D deficiency or lack of calcium.**<br><br>**Leads to weak and soft bones that can bend or bow.**<br><br>**Blount's Disease:**<br><br>**A growth disorder affecting the shin bone (tibia).**<br><br>**Causes abnormal growth at the top of the shin bone, leading to progressive bowing.**<br><br>**More common in early walkers and overweight children.**<br><br>**Bone Dysplasia (Genetic Disorders):**<br><br>**Conditions like achondroplasia (a type of dwarfism) can cause bowlegs.**<br><br>**Fractures or Bone Injuries:**	• Usually disappears on its own by age 3 or 4.<br><br>**2. Blount's Disease:**<br><br>• This condition affects the growth plate in the shinbone (tibia), causing the bone to grow unevenly and leading to bowing.<br>• More common in children who start walking early or are overweight.<br>• Early diagnosis is important to prevent progressive bowing.<br><br>**3. Rickets:**<br><br>• Caused by a deficiency of vitamin D, which is essential for bone health.<br>• Leads to soft and weak bones, making them more prone to bending.<br>• Can be prevented with adequate	D, calcium, or phosphate, leading to weak and soft bones. Rickets can cause bowlegs or worsen the condition.<br>3. **Blount's disease:** A growth disorder affecting the tibia (shinbone), causing the bone to grow abnormally and resulting in bowlegs. This condition often requires medical intervention.<br>4. **Bone infections or fractures:** Infections or injuries to the bones can affect their growth and alignment, potentially leading to bowlegs.<br>5. **Genetic factors:** In some cases, bowlegs can be inherited from parents who have the condition.<br><br>If you're concerned about a child's leg alignment or if the bowlegs persist beyond early

	**Poor healing after fractures can result in one leg bowing.**<br><br>**Infections or Inflammation:**<br><br>**Certain infections affecting bone growth can cause bowing.**<br><br>⚠️ **Risk Factors:**<br><br>**Early walking: Putting weight on legs before bones fully develop.**<br><br>**Obesity: Extra weight can stress leg bones, worsening bowing.**<br><br>**Family history: Genetic predisposition to bone or growth disorders.**	vitamin D intake and sun exposure.<br><br>**4. Skeletal Dysplasias:**<br><br>• This is a group of genetic conditions that affect bone growth and development.<br>• Can cause various skeletal abnormalities, including bowlegs.<br>• Achondroplasia is a relatively common type of skeletal dysplasia.<br><br>**5. Injury or Fracture:**<br><br>• A previous injury or fracture in the leg can sometimes disrupt bone growth and lead to bowing.<br><br>**6. Other less common causes:**<br><br>• Lead poisoning<br>• Fluoride poisoning	childhood, it's best to consult with a healthcare professional to determine the underlying cause and appropriate treatment.

		• Bone infections<br><br>**Important to remember:**<br><br>• The underlying cause of bowlegs can vary depending on the child's age, the severity of the bowing, and other associated symptoms.<br>• If you have any concerns about your child's leg development, it's crucial to consult with their doctor. They can perform a thorough evaluation to determine the cause and recommend the best course of action.<br><br>Sources and related content	
4. How do I know if my child's	Signs That Bowlegs Are Normal:<br><br>1. **Age:**	t's great you're paying close attention to your child's leg development! Here's how to	the following:

<table>
<tr>
<td rowspan="2">bowlegs are normal or need a doctor’s help?</td>
<td>o Common and normal in infants and toddlers up to age 2–3.
o Often corrects itself by age 4–5.
2. Both legs bowed symmetrically:
o Even, gradual improvement as they grow.
3. No pain or difficulty walking:
o Child walks, runs, and plays without limping or discomfort.
4. No unusual symptoms:
o No redness, swelling, or fever around the knees.</td>
<td rowspan="2">tell if their bowlegs might be something to discuss with your doctor:

Generally Normal (Especially in Younger Children):

• Mild bowing: A slight outward curve of the legs is common in babies and toddlers.
• Symmetrical: Both legs are bowed to about the same degree.
• Improves with age: The bowing gradually lessens as your child grows, especially between ages 2 and 4.
• No other symptoms: Your child walks, runs, and plays without pain or difficulty.

When to See a Doctor:

• Severe bowing: The outward curve is very</td>
<td rowspan="2">1. Age: Bowlegs are common in infants and toddlers. If your child is under 3 years old and the bowlegs are symmetrical (both legs curve outward equally), it's usually a normal part of development.
2. Improvement over time: If your child's bowlegs are gradually improving as they grow, it's likely a normal variation. Bowlegs often correct themselves by the age of 3 to 4 years.
3. Severity: If the bowlegs are severe, meaning the gap between the knees is large when the ankles are together, it may be worth consulting a healthcare professional.
4. Pain or discomfort: If your child is experiencing pain, discomfort, or</td>
</tr>
<tr>
<td>⚠ Signs You Should See a Doctor:

1. Age Concern:
o Bowlegs persist beyond age 3–4 without improvement.
2. Severity and Worsening:
o Bowing is severe or seems to be getting worse instead of improving.
3. Asymmetrical Bowing:
o Only one leg is bowed or one is</td>
</tr>
</table>

	significantly worse than the other.<br>4. **Pain or Walking Issues:**<br>o Child has **knee pain, limp, or difficulty running**.<br>5. **Family History:**<br>o Family history of bone disorders or conditions like **Blount's disease**.<br>6. **Unusual Symptoms:**<br>o **Short stature, bone pain, or frequent fractures**.<br><br>When Visiting a Doctor:<br><br>• They might do a **physical exam, X-rays, or blood tests** (to check for rickets or other conditions).<br>• Early diagnosis can lead to more effective treatment if needed.	noticeable or seems to be getting worse.<br>• **Asymmetrical bowing:** One leg is significantly more bowed than the other.<br>• **Doesn't improve with age:** The bowing persists or worsens beyond age 3 or 4.<br>• **Pain or discomfort:** Your child complains of pain in their legs or knees, or they limp or have difficulty walking or running.<br>• **Other symptoms:** You notice any other unusual symptoms, such as swelling, stiffness, or changes in skin color in the legs or feet.<br>• **Family history:** There's a family history of bone diseases or skeletal problems.<br><br>**Important Notes:**	difficulty walking, it's important to seek medical advice.<br>5. **Asymmetry:** If one leg is more curved than the other, or if there are noticeable differences in leg length or alignment, it's advisable to consult a doctor.<br>6. **Growth patterns:** If the bowlegs persist or worsen beyond the age of 3 to 4 years, or if your child shows other signs of growth abnormalities, a doctor's evaluation is recommended.<br>7. **Family history:** If there's a family history of conditions like rickets or Blount's disease, it's a good idea to discuss your concerns with a healthcare professional.<br><br>If you're unsure or concerned about your child's

		Early intervention is key: If there is an underlying condition causing the bowlegs, early diagnosis and treatment can make a big difference. **Trust your instincts:** If you have any concerns at all, it's always best to err on the side of caution and talk to your doctor. They can examine your child, answer your questions, and provide reassurance or recommend any necessary evaluations. <br>I hope this helps! Let me know if you have any other questions.<br><br>Sources and related content	leg alignment, it's always best to consult with a pediatrician or orthopedic specialist. They can assess your child's condition, provide guidance, and recommend any necessary treatments. Your child's health and well-being are paramount, so don't hesitate to seek professional advice if you have any doubts.
5. What tests do doctors	When you visit a doctor to check for **bowlegs** in your child, they might use a combination of **physical exams,**	Doctors use a combination of approaches to assess bowlegs	Doctors use several tests to diagnose bowlegs and determine if there's an

use to check for bowlegs?	**imaging tests, and sometimes lab tests** to understand the cause and severity. Here's what to expect:<br><br>1. Physical Examination:<br><br>• **Visual inspection:** The doctor will ask your child to stand straight with feet together to observe leg alignment.<br>• **Measuring the gap:** They may measure the distance between the knees when the ankles are together.<br>• **Gait analysis:** Observing how your child walks to check for limping or uneven stride.<br><br>2. Imaging Tests:<br><br>1. **X-rays:**<br>o Most common test for bowlegs.<br>o Helps detect bone deformities, measure the angle of bowing, and identify conditions like **Blount's disease** or fractures.<br>2. **MRI or CT Scan:** *(Rarely needed)*	in children. Here are the common methods:<br><br>**1. Physical Exam:**<br><br>• **Visual assessment:** The doctor will observe the child's legs while they're standing and walking to assess the degree and symmetry of bowing.<br>• **Measuring the space between the knees:** With the child standing and their ankles touching, the doctor will measure the distance between the knees. This helps quantify the severity of the bowing.<br>• **Checking range of motion:** The doctor will examine the joints in the legs to ensure they have a normal range of motion.<br><br>**2. X-rays:**	underlying condition. Here are some common tests and evaluations:<br><br>1. **Physical examination:** The doctor will visually inspect the child's legs and measure the distance between the knees while the child is lying on their back. They will also observe how the child walks to identify any abnormalities.<br>2. **Blood tests:** These tests can help identify deficiencies in vitamin D, calcium, or phosphate, which are common causes of rickets2.<br>3. **X-rays:** Imaging tests like X-rays are used to examine the bones in the legs and knees. This helps to identify any structural issues or

	◦ Used if more detailed images are required, especially for joint or bone abnormalities.<br><br>3. Blood Tests: *(If a metabolic or nutritional cause is suspected)*<br><br>• **Vitamin D, calcium, and phosphorus levels:** To check for **rickets**.<br>• **Alkaline phosphatase:** Can be elevated in bone disorders.<br><br>4. Angle Measurement:<br><br>• **Intermalleolar or Intercondylar distance:**<br>◦ Measures the space between the knees or ankles when standing.<br>• **Tibiofemoral angle:** To assess the degree of bowing.	• **Bone alignment:** X-rays provide detailed images of the bones, allowing the doctor to assess the alignment of the leg bones and identify any underlying structural abnormalities.<br>• **Growth plates:** X-rays can also show the growth plates, which are areas of cartilage at the ends of long bones where growth occurs. This is particularly important in diagnosing conditions like Blount's disease.<br><br>**3. Other tests (if needed):**<br><br>• **Blood tests:** May be done to check for vitamin D deficiency (rickets) or other metabolic conditions.<br>• **Genetic testing:** May be considered if a	growth abnormalities2.<br>4. **MRI:** In some cases, an MRI may be performed to get a more detailed view of the bones and soft tissues.<br><br>These tests help doctors determine the cause of bowlegs and decide on the appropriate treatment. If you have concerns about your child's leg alignment, it's best to consult with a healthcare professional for a thorough evaluation.

		skeletal dysplasia is suspected.<br><br>**Important points:**<br><br>• The type and extent of testing will depend on the child's age, the severity of bowing, and any other symptoms they may have.<br>• In many cases, especially with young children who have mild bowing, a physical exam may be sufficient.<br>• X-rays are often used when the bowing is significant, asymmetrical, or doesn't improve with age.<br><br>If you're concerned about your child's legs, it's always best to consult with their doctor. They can determine the appropriate tests and provide you with	

		personalized advice and guidance.<br><br>Sources and related content	
6. Will bowlegs get better on their own as my child grows?	✅ When Bowlegs Improve Naturally:<br><br>• **Infants and Toddlers:**<br>○ Bowlegs are normal up to **age 2–3** due to their fetal position in the womb.<br>○ As children begin to walk and grow, their legs usually straighten naturally.<br>• **By Age 4–5:**<br>○ Most children's legs have either straightened or temporarily shifted to a **knock-kneed** (knees touch, ankles apart) phase, which is also normal.<br>• **By Age 7–8:**<br>○ Legs typically reach permanent alignment without any treatment.	It's great that you're paying attention to your child's development! Whether bowlegs get better on their own really depends on what's causing them. Here's the breakdown:<br><br>**Most likely, YES, they will get better:**<br><br>• **Physiologic bowing:** This is the most common type in young children, and it usually improves naturally as they grow. By age 3 or 4, most kids with physiologic bowing will have straight legs.	In many cases, bowlegs will naturally improve as a child grows. Here's what to expect:<br><br>• **Infants and toddlers:** Bowlegs are common in babies due to their fetal position in the womb. As they begin to walk and bear weight on their legs, the condition may become more noticeable but often corrects itself over time.<br>• **Preschool age:** By the age of 3 to 4 years, most children's legs will have straightened out. If the bowlegs persist beyond this age, it

<table>
<tr>
<td></td>
<td>
⚠ When Bowlegs Might Not Improve on Their Own:

Severe bowing or bowing that worsens after age 2–3.
Asymmetrical bowing: One leg is more bowed than the other.
Medical conditions:

Blount’s Disease: Causes progressive bowing if untreated.
Rickets: Due to vitamin D deficiency, may require supplements.
Genetic bone disorders: Such as achondroplasia.

Pain or difficulty walking: Indicates a need for evaluation.

<hr>
🛠 Treatment Options if Needed:

Braces or orthotics: For mild to moderate cases.
Surgery: In severe or progressive cases (usually delayed until after age 10 if possible).

</td>
<td>
Possibly, but needs monitoring:

Blount's disease: Early diagnosis and treatment are key. With proper bracing or other interventions, many children with Blount's disease can see improvement and avoid surgery.

May need treatment to improve:

Rickets: With vitamin D supplementation and correction of any underlying nutritional deficiencies, the bowing can often improve, especially if caught early.

Less likely to improve without intervention:

Skeletal dysplasias: These genetic conditions often require ongoing

</td>
<td>
may be a sign of an underlying condition that requires medical attention.
However, it's essential to monitor your child's development. If the bowlegs are severe, cause pain or discomfort, or if there are noticeable differences between the legs, it's best to consult with a healthcare professional. They can evaluate your child's condition and recommend any necessary treatments to ensure healthy growth and development.
</td>
</tr>
</table>

	• **Nutritional supplements:** For rickets (vitamin D and calcium).	management and may not completely resolve on their own.<br>• **Injury or fracture:** Depending on the severity and location of the injury, some bowing may persist.<br><br>**Here's what you can do:**<br><br>• **Regular checkups:** Take your child for well-child visits so their doctor can monitor their growth and development, including their leg alignment.<br>• **Address any concerns:** If you notice anything unusual or have any worries about your child's legs, don't hesitate to talk to their doctor.<br>• **Provide a healthy lifestyle:** Ensure your child gets enough vitamin D, calcium, and other nutrients	

		essential for bone health. Encourage physical activity to support strong bones and muscles.<br><br>Ultimately, the best way to know if your child's bowlegs will improve on their own is to have them evaluated by a pediatrician. They can determine the cause of the bowing and advise you on the best course of action.	
7. What treatments are available for bowlegs?	he **treatment for bowlegs** depends on the cause, severity, and the child's age. In many cases, especially for young children, no treatment is needed as the legs straighten naturally. However, if treatment is required, here are the main options:<br><br>☑ 1. Observation and Monitoring:<br><br>- **For infants and toddlers:**<br>○ Bowlegs are usually normal up to **age 2–3** and often correct themselves by age 4–5.	The treatment for bowlegs depends on several factors, including the cause, the severity of the bowing, and the child's age. Here are some common treatment options:<br><br>**1. Observation:**<br><br>- **For physiologic bowing:** In most cases, especially in young children with mild bowing, no active treatment is needed. The doctor	There are several treatment options available for bowlegs, depending on the underlying cause and the severity of the condition. Here are some common treatments:<br><br>1. **Observation:** For infants and toddlers, bowlegs often correct themselves as the child grows. Regular monitoring by a healthcare professional is usually sufficient.

	◦ Regular check-ups to monitor improvement.<br>• **When it’s enough:**<br>◦ If bowing is **mild, symmetrical, and improving with age**.<br><br>2. Braces and Orthotics:<br><br>• **Leg braces or splints:**<br>◦ Used for conditions like **Blount’s disease** to guide proper growth.<br>◦ Often worn at night or for several hours a day.<br>• **Special shoes or orthotics:**<br>◦ Provide support but have limited effectiveness for structural bowing.<br><br>3. Nutritional Supplements:<br><br>• **For rickets:**<br>◦ **Vitamin D and calcium supplements** to strengthen bones.<br>◦ Dietary changes (fortified milk, fish,	will simply monitor the child's growth and development to ensure the bowing is improving as expected.<br><br>**2. Bracing:**<br><br>• **For Blount's disease:** Bracing is often the first line of treatment for Blount's disease, especially in younger children. The brace helps to guide the growth of the shinbone and correct the bowing.<br>• **KAFO (knee-ankle-foot orthosis):** This type of brace is commonly used for Blount's disease. It extends from the thigh to the foot and helps to realign the leg.<br><br>**3. Surgery:**<br><br>• **For severe or persistent bowing:**	2. **Nutritional supplements:** If bowlegs are caused by rickets, supplements of vitamin D, calcium, or phosphate can help strengthen the bones and correct the condition.<br>3. **Bracing:** In some cases, especially for children with Blount's disease, leg braces can help guide the bones into proper alignment as they grow.<br>4. **Physical therapy:** Exercises to strengthen the muscles around the knees and improve leg alignment can be beneficial, particularly for mild cases.<br>5. **Surgery:** In severe cases or when other treatments are ineffective, surgical options may be

<table>
<tr>
<td rowspan="3"></td>
<td>or sunlight exposure).</td>
<td rowspan="3">Surgery may be considered if bracing is unsuccessful or if the bowing is severe and causing significant problems.
• Osteotomy: This is the most common surgical procedure for bowlegs. It involves cutting and realigning the bone to correct the deformity.
• Guided growth: In some cases, a surgical technique called guided growth may be used. This involves placing temporary plates or screws across the growth plate to guide the bone's growth in the desired direction.

4. Other treatments:

• Vitamin D supplementation: For rickets, vitamin D supplements are</td>
<td rowspan="3">considered. These can include:
o Guided growth: A small metal plate or screws are inserted at the growth plate to gradually correct the alignment as the child grows.
o Osteotomy: This procedure involves cutting and realigning the bones to correct the bowlegs.
o Arthroscopy: In some cases, minimally invasive surgery can be used to address joint issues related to bowlegs.</td>
</tr>
<tr>
<td>4. Surgery:

For severe or persistent bowlegs (usually delayed until after age 10 if possible):

1. Guided Growth Surgery (Hemiepiphysiodesis):
o Metal plates or staples temporarily placed on one side of the growth plate to allow the other side to catch up.
o Minimally invasive and effective for growing children.
2. Osteotomy:
o Bone is cut and realigned to correct the bowing.
o Often needed for older children or severe cases.
3. External Fixation:
o A frame outside the leg is used to gradually straighten the bone.</td>
</tr>
<tr>
<td></td>
</tr>
</table>

	⚖️ 5. Physical Therapy:<br><br>• **Exercises:** Strengthen leg muscles and improve walking patterns.<br>• **Stretching:** Helps if bowing causes muscle tightness.<br><br>⚠ When to Consider Treatment:<br><br>• **Bowing gets worse after age 3.**<br>• **Asymmetrical bowing** (one leg more bowed).<br>• **Pain, limping, or difficulty walking.**<br>• **Underlying medical condition** like rickets or Blount's disease.	crucial to strengthen the bones and improve bowing.<br>• **Nutritional support:** Ensuring adequate calcium and other nutrients is important for bone health and may aid in the treatment of bowlegs.<br>• **Physical therapy:** In some cases, physical therapy may be recommended to improve muscle strength, flexibility, and range of motion.<br><br>**Important considerations:**<br><br>• **Early diagnosis and treatment are crucial:** This is especially true for conditions like Blount's disease, where early intervention can often prevent the need for surgery.<br>• **Treatment plans are individualized:** The	6. **Lifestyle modifications:** For adults with bowlegs, maintaining a healthy weight and engaging in low-impact exercises can help manage symptoms and prevent further complications.<br><br>If you have concerns about bowlegs or think treatment might be necessary, it's best to consult with a healthcare professional for a thorough evaluation and personalized recommendations.

		best treatment approach will vary depending on the child's specific needs and circumstances.<br>• **Regular follow-up is essential:** To monitor progress and make any necessary adjustments to the treatment plan.<br><br>If you're concerned about your child's bowlegs, it's important to consult with their doctor. They can evaluate the condition, determine the underlying cause, and recommend the most appropriate treatment options.	
8. What problems might happen if bowlegs are not treated?	If **bowlegs** are not treated when necessary, especially in cases where they are severe or caused by underlying conditions, it can lead to **long-term complications**. Here are some potential problems:<br><br>1. Knee and Joint Problems:<br><br>• **Osteoarthritis:**	It's important to address bowlegs, especially when they are severe or caused by an underlying condition. Here are some potential problems that can arise if bowlegs are left untreated:	f bowlegs are not treated, several complications can arise over time. Some of the potential problems include:<br><br>1. **Arthritis:** Untreated bowlegs can lead to uneven stress on the knee and hip joints, increasing the risk of

<table>
<tr>
<td rowspan="2"></td>
<td>

<li style="list-style:none">

Uneven weight distribution can lead to early wear and tear on the inner part of the knee joint.
Increases the risk of arthritis in the 20s or 30s instead of later in life.

Meniscus tears:

Extra stress on the knees can cause cartilage damage or tears.

Patellar tracking issues:

Misalignment can cause the kneecap to move improperly, leading to pain and instability.

</td>
<td rowspan="2">
1. Difficulty with movement and mobility:

Awkward gait: Bowlegs can affect a child's walking pattern, leading to an awkward or unsteady gait. This can make it harder to keep up with peers and participate in physical activities.
Limited range of motion: Severe bowing can restrict the range of motion in the knees and ankles, making certain movements difficult or painful.

2. Pain and discomfort:

Knee pain: The abnormal alignment of the legs can put extra stress on the knee joints, leading to pain and discomfort, especially during or after physical activity.

</td>
<td rowspan="2">
developing arthritis, particularly in the knees and hips.
<ol start="2">
Gait abnormalities: Bowlegs can affect posture and movement efficiency, leading to an abnormal walking pattern. This can cause discomfort and difficulty in physical activities.
Lower back pain: The altered alignment of the legs can affect the spine, leading to lower back pain due to compensatory postural adjustments.
Joint instability: Bowlegs can cause joint instability, making physical activities more challenging and increasing the risk of injuries.
Muscle imbalances: The body may compensate for the misalignment,

</td>
</tr>
<tr>
<td>
2. Gait and Mobility Issues:

Limping or waddling gait:

Severe bowing alters the way a child walks, which can cause muscle imbalances and fatigue.

Reduced athletic ability:

Difficulty running, jumping, or balancing properly.

</td>
</tr>
</table>

	3. Bone and Growth Problems:<br><br>• **Leg length discrepancy:**<br>○ If only one leg is bowed, it can cause **one leg to appear shorter** than the other.<br>• **Progressive worsening:**<br>○ Conditions like **Blount's disease** can cause bowing to worsen without treatment.<br><br>4. Pain and Discomfort:<br><br>• **Knee, hip, or ankle pain:**<br>○ Misalignment puts extra stress on these joints, causing **chronic pain** over time.<br>• **Lower back pain:**<br>○ Abnormal posture and gait can strain the back.<br><br>5. Social and Emotional Impact:<br><br>• **Self-consciousness or bullying:**	• **Hip pain:** In some cases, bowlegs can also affect the hips, causing pain in that area.<br><br>**3. Joint problems later in life:**<br><br>• **Osteoarthritis:** Untreated bowlegs can increase the risk of developing osteoarthritis in the knees and hips later in life due to the added wear and tear on the joints.<br><br>**4. Psychological impact:**<br><br>• **Self-consciousness:** Children with noticeable bowlegs may feel self-conscious about their appearance, which can affect their self-esteem and social interactions.<br>• **Bullying:** Unfortunately, children with	leading to muscle imbalances that can affect overall mobility and function.<br>6. **Increased risk of injury:** Athletes and active individuals with untreated bowlegs may be more prone to injuries due to the uneven distribution of stress on the joints and muscles.<br><br>It's important to address bowlegs, especially if they persist beyond early childhood or cause discomfort, to prevent these complications and ensure healthy growth and development. If you have concerns about bowlegs, consulting with a healthcare professional is the best course of action.

- Visible bowing can affect self-esteem, especially in older children and teens.
- **Avoidance of physical activities:**
  - Pain or embarrassment might discourage sports or play.

Key Takeaway:

- **Mild bowlegs** often correct naturally and don’t cause problems.
- **Moderate to severe cases** or bowlegs due to underlying conditions might need **treatment** to avoid these complications.

physical differences can sometimes be targets of bullying, which can have a negative impact on their emotional well-being.

5. Complications specific to underlying causes:

- **Blount's disease:** If left untreated, Blount's disease can lead to progressive bowing, limb length discrepancy (one leg shorter than the other), and severe joint damage.
- **Rickets:** Untreated rickets can cause bone deformities, delayed growth, and other health problems.

Important note:

- The severity of potential problems varies depending on

		the cause and severity of the bowlegs.<br>• Early diagnosis and treatment are crucial to minimize the risk of long-term complications.<br><br>If you have any concerns about your child's bowlegs, it's essential to consult with their doctor. They can assess the condition, determine the need for treatment, and help prevent potential problems in the future.<br><br>Sources and related content	
9. Do braces or other treatments work for bowlegs?	Yes, **braces and other treatments can work for bowlegs**, especially for children whose bones are still growing. The effectiveness depends on the **cause, severity, and timing** of the treatment. Here's a breakdown:	Yes, braces and other treatments can be effective for bowlegs, especially when implemented early and for specific causes. Here's how they work:<br><br>**Braces:**<br><br>• **How they work:** Braces, like the	children. The success of these treatments depends on the underlying cause of the bowlegs and the child's age. Here are some commonly used treatments:<br><br>1. **Bracing:** In children with conditions like Blount's disease, leg braces can help guide

	1. Braces and Orthotics:<br><br>**Effectiveness:** ⭐⭐⭐☆ (Works well for certain conditions if started early)<br><br>• **When they help:**<br>○ Effective for mild to moderate cases and for conditions like **Blount's disease**.<br>○ Most beneficial when used **before age 4** while bones are still developing.<br>• **Types:**<br>○ **Knee-ankle-foot orthoses (KAFO):** Braces that guide the legs to grow straighter.<br>○ **Night braces:** Worn during sleep to gradually correct alignment.<br>• **Limitations:**<br>○ Less effective for severe cases or if bowing is due to **rickets** or genetic disorders.<br><br>🌞 2. Nutritional Supplements:	KAFO (knee-ankle-foot orthosis), apply gentle pressure to guide the growth of the leg bones. They are most effective for Blount's disease in young children (under 4 years old) when the growth plates are still open.<br>• **Success rates:** Bracing can successfully correct bowing in many cases of Blount's disease, potentially avoiding the need for surgery. The earlier the treatment starts, the better the chances of success.<br><br>**Surgery:**<br><br>• **Osteotomy:** This procedure involves cutting and realigning the bone. It's often used for severe bowing or when bracing is not effective.	the bones into proper alignment as they grow. Braces are most effective when used early, while the bones are still developing.<br>2. **Nutritional supplements:** If bowlegs are caused by rickets, supplements of vitamin D, calcium, or phosphate can help strengthen the bones and correct the condition.<br>3. **Physical therapy:** Exercises to strengthen the muscles around the knees and improve leg alignment can be beneficial, particularly for mild cases of bowlegs.<br>4. **Surgery:** In severe cases or when other treatments are ineffective, surgical options may be considered. These can include:

	**Effectiveness:** ⭐⭐⭐⭐☆ (Very effective for rickets)<br><br>• **When they help:**<br>○ Work well if bowlegs are caused by **rickets** (vitamin D and calcium deficiency).<br>• **Treatment:**<br>○ **Vitamin D, calcium, and phosphorus** supplements.<br>○ Sunlight exposure and diet changes.	• **Guided growth:** This technique uses temporary plates or screws to guide bone growth. It may be an option for younger children with specific types of bowing.<br>• **Success rates:** Surgical treatments generally have high success rates in correcting bowlegs, but like any surgery, they carry some risks and require a recovery period.<br><br>**Other Treatments:**<br><br>• **Vitamin D supplementation:** Essential for treating rickets, vitamin D helps strengthen bones and can improve bowing caused by this deficiency.<br>• **Nutritional support:** Ensuring adequate calcium and other nutrients is important	○ **Guided growth:** A small metal plate or screws are inserted at the growth plate to gradually correct the alignment as the child grows.<br>○ **Osteotomy:** This procedure involves cutting and realigning the bones to correct the bowlegs.<br>5. **Observation:** For infants and toddlers, bowlegs often correct themselves as the child grows. Regular monitoring by a healthcare professional is usually sufficient.<br><br>It's important to consult with a healthcare professional to
	🛠️ 3. Surgery:<br><br>**Effectiveness:** ⭐⭐⭐⭐⭐ (Highly effective for severe cases)<br><br>• **When it's needed:**<br>○ For **severe, worsening, or asymmetrical** bowlegs, or if caused by conditions like **Blount's disease**.<br>• **Types:**<br>○ **Guided Growth Surgery:**<br>▪ Temporary metal plates or			

	staples placed to guide bone growth.<br>▪ Minimally invasive and effective for growing children.<br>o **Osteotomy:**<br>▪ Bone is cut and realigned to correct severe bowing.<br>o **External Fixation:**<br>▪ An external frame gradually straightens the bone.<br><br>⚖️ 4. Physical Therapy:<br><br>**Effectiveness:** ⭐⭐☆☆ (Supportive, not corrective)<br><br>• **When it helps:**<br>o Strengthens muscles around the knees and improves **gait and balance**.<br>• **Exercises:**	for overall bone health and can support the effectiveness of other treatments.<br><br>**Factors influencing treatment success:**<br><br>• **Cause of bowlegs:** Treatments are most effective when tailored to the underlying cause.<br>• **Age of the child:** Early intervention is often key, especially for conditions like Blount's disease, where treatment is most effective while the bones are still growing.<br>• **Severity of bowing:** Mild bowing may improve with observation or minimal intervention, while severe bowing might require more intensive treatment.<br>• **Compliance with treatment:**	determine the most appropriate treatment for your child's specific situation. Early intervention can help ensure the best possible outcome and prevent potential complications.

	◦ Stretching, leg lifts, and balance activities.<br><br>Key Takeaway:<br>• **Braces:** Work best for **mild to moderate** bowlegs if started early.<br>• **Surgery:** Most effective for **severe cases** or older children.<br>• **Supplements:** Essential for **rickets-related bowlegs**.	Consistent use of braces or adherence to other treatment recommendations is crucial for optimal results.<br><br>It's important to remember that every child is different, and treatment outcomes can vary. Consulting with a doctor or orthopedic specialist is essential to determine the most appropriate and effective treatment plan for your child's specific needs.	

Table (2) : shows the questions about Knock knees and responses among the three chatbots:

Question	Chatgpt	Geminini	Copilot
10. What are knock knees?	Knock knees, or **genu valgum**, is a condition where a person's knees touch when their feet are apart. This can create a noticeable inward angle of the legs. It is common in young children and often corrects itself with growth, but in some cases, it may persist into adulthood.	Knock knees, also known as genu valgum, is a condition where the knees angle inward and touch each other when the legs are straightened.	Knock knees, medically known as genu valgum, is a condition where a person's knees touch, but their ankles do not. This alignment causes the knees to angle inward while standing or walking. It's most common in

<table>
<tr>
<td></td>
<td>
Causes of Knock Knees

1. Normal growth and development – Most children aged 2-6 have mild knock knees that resolve naturally by age 7.
2. Genetics – Some people inherit a tendency for knock knees.
3. Underlying medical conditions, such as:
o Rickets (caused by vitamin D deficiency)
o Obesity (excess weight can stress the knee joints)
o Injuries or fractures
o Arthritis (especially in older adults)

Symptoms

• Knees touch while standing with feet apart
• Difficulty walking or discomfort in severe cases
• Uneven wear on shoes due to altered leg alignment

Treatment Options
</td>
<td>
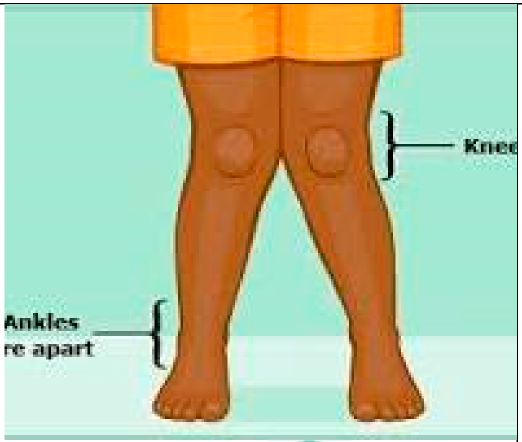

Opens in a new window
kidshealth.org
Knock knees (genu valgum)

This can cause the legs to appear bowed outward below the knees. In most cases, it's a normal variation in children and tends to correct itself as they grow. However, in some cases, it can be a sign of an underlying medical condition.

Causes:

• Physiological genu valgum: This is the most common type and is considered a normal part of development in young children. It usually resolves on its own by the age of 7 or 8.
• Nutritional deficiencies: Rickets, a condition caused by vitamin D deficiency, can lead to knock knees.
</td>
<td>
children between the ages of 2 and 5, usually correcting itself as they grow. However, if it persists beyond early childhood, it might be due to underlying issues like vitamin D deficiency, genetics, or other medical conditions.

Common symptoms include:

• Visible inward bending of the knees.
• Difficulty walking or running.
• Knee pain or discomfort.

Treatment options can range from simple observation (as many cases resolve naturally) to exercises, orthotics, and in rare cases, surgery. Consulting a healthcare professional is the
</td>
</tr>
</table>

	• **Observation** (for young children, as it often resolves naturally)<br>• **Exercise & physical therapy** (to strengthen leg muscles)<br>• **Bracing** (in growing children with more severe cases)<br>• **Surgery** (in extreme or painful cases, especially in adults)	• **Bone diseases:** Conditions like Blount's disease or bone dysplasia can affect bone growth and cause knock knees.<br>• **Injuries:** Trauma to the knee joint can sometimes result in knock knees.<br>• **Obesity:** Excess weight can put stress on the knees and contribute to the development of knock knees.<br>• **Genetic conditions:** In rare cases, knock knees can be inherited.<br><br>**Symptoms:**<br><br>• Knees touching when standing with feet together<br>• Outward angling of the lower legs<br>• Difficulty walking or running<br>• Knee pain<br>• Instability<br><br>**Treatment:**<br><br>Treatment for knock knees depends on the underlying cause and the severity of the condition. In most cases, physiological genu valgum resolves on its own and requires no treatment. However, if the condition is severe or caused by an underlying medical condition, treatment options may include:<br><br>• **Observation:** Regular checkups to monitor the condition.<br>• **Bracing:** Wearing a brace to help straighten the legs.	best way to determine the right approach if you or someone you know is experiencing this.<br><br>Feel free to ask more if you have further questions!<br><br>What causes knock knees?How are knock knees diagnosed?What treatments are available for knock knees?<br><br>.

		• **Physical therapy:** Exercises to strengthen the muscles around the knees.<br>• **Surgery:** In severe cases, surgery may be necessary to correct the alignment of the bones.<br><br>If you're concerned about knock knees, it's important to see a doctor for diagnosis and treatment.<br><br>or pediatrician for proper evaluation and guidance. They can determine the underlying cause and recommend the most appropriate treatment options.	
11. When is it normal for a child to have knock knees?	It is **normal** for children to have knock knees between the ages of **2 and 6**. This is part of natural growth and development as their legs adjust to bearing weight and walking.<br><br>Typical Development of Leg Alignment in Children:<br><br>• **Birth to 18 months:** Babies often have **bowed legs** (**genu varum**), which is normal due to their fetal position in the womb.<br>• **18 months to 2 years:** The legs start straightening.	t's common for children to go through phases of leg alignment changes as they grow. Here's a breakdown of when knock knees are typically considered normal:<br><br>• **Normal developmental phase:**<br>○ Knock knees are most commonly observed in children between the ages of 3 and 5.<br>○ It's a normal part of their growth and development, and in most cases, it resolves on its own.<br>○ Generally, the knock-kneed appearance tends to peak around age 4.<br>○ By the age of 7 or 8, the legs usually straighten out.<br>• **When to seek medical advice:**	It's quite common for young children to have knock knees. Here's a general timeline:<br><br>• **Toddlers (ages 2-3)**: It's normal for knock knees to develop as they start walking. Their knees might appear to angle inward, which is a part of the

	• **2 to 4 years:** Many children develop mild **knock knees (genu valgum)** as part of normal growth.<br>• **4 to 7 years:** The knees gradually straighten, and the leg alignment typically becomes normal.<br><br>By **age 7**, most children’s legs should have a straight or slightly outward alignment. If knock knees persist beyond this age, worsen, or cause pain, a doctor should evaluate them.<br><br>Would you like advice on when to seek medical attention?	o If knock knees appear before the age of 2 or worsen after the age of 7, it's advisable to consult a doctor.<br>o Also, seek medical advice if:<br>▪ The knock knees are severe.<br>▪ The child experiences pain or difficulty walking.<br>▪ Only one leg is affected.<br>▪ The child has other symptoms, such as short stature.<br><br>In essence, knock knees are often a normal part of childhood development, but it's essential to be aware of when they might indicate a more serious underlying condition.<br><br>Sources and related content	normal growth process.<br>• **Preschoolers (ages 3-5)**: Knock knees are often most pronounced around this age. However, as children grow and their muscles and bones develop, the condition typically begins to correct itself.<br>• **School-age children (ages 6-7)**: By this age, most children naturally outgrow knock knees, and their leg alignment becomes more straight. If

			the condition persists beyond this age, it might be a good idea to consult a healthcare professional to rule out any underlying issues.<br><br>So, it's quite normal for children to have knock knees between the ages of 2 and 7. If you have any concerns, talking to a doctor can provide peace of mind and guidance on any needed treatments.<br><br>What causes knock knees in children?How common are knock knees and how can they be treated?What signs indicate I should consult a doctor?

12. What can cause knock knees in a child?	Knock knees (**genu valgum**) in children can be caused by several factors, ranging from normal growth patterns to underlying medical conditions.<br><br>Common Causes of Knock Knees in Children:<br><br>1. Normal Growth and Development<br><br>• Most children between ages **2 and 6** develop mild knock knees as part of their natural growth.<br>• Usually corrects itself by age **7** without treatment.<br><br>2. Nutritional Deficiencies<br><br>• **Rickets** (caused by vitamin D, calcium, or phosphate deficiency) can weaken bones, leading to knock knees.<br>• More common in children with poor nutrition or limited sun exposure.<br><br>3. Obesity<br><br>• Excess weight can put extra stress on the knee	Knock knees in children can arise from a few different factors, and it's important to differentiate between normal developmental changes and potential underlying medical issues. Here's a breakdown:<br><br>**Common Causes:**<br><br>• **Normal Development:**<br>o This is the most frequent cause. Many children naturally go through a phase where their legs angle inward, typically between ages 3 and 5. This is usually a temporary condition that corrects itself as they grow, generally by age 7 or 8.<br>• **Medical Conditions (Less Common):**<br>o **Rickets:**<br>▪ This is a condition caused by a deficiency in vitamin D, calcium, or phosphate, leading to weakened bones.<br>o **Bone Dysplasia:**<br>▪ These are disorders that affect bone growth and development.<br>o **Blount's Disease:**<br>▪ This is a growth disorder that affects the growth plate in the upper part of	Knock knees in children can be caused by several factors, including:<br><br>1. **Normal growth and development**: It's common for children between the ages of 2 and 7 to have knock knees as part of their natural growth process. Most children outgrow this condition as they develop.<br>2. **Genetic conditions**: Some children may inherit skeletal dysplasias or other

	joints, affecting leg alignment.<br><br>4. Genetic Factors<br><br>• Some children inherit a tendency for knock knees from their parents.<br><br>5. Bone or Joint Conditions<br><br>• **Blount's disease** – A growth disorder affecting the shinbone (**tibia**), causing severe knock knees.<br>• **Osteogenesis imperfecta** – A genetic condition leading to weak and brittle bones.<br>• **Arthritis** (though rare in young children) can cause joint deformities, including knock knees.<br><br>6. Previous Injuries or Fractures<br><br>• A fracture that heals incorrectly can lead to misalignment of the legs.<br><br>When to Seek Medical Help<br><br>• Knock knees persist **beyond age 7**.<br>• The condition is **severe or worsening** over time.	the shinbone (tibia).<br>o **Injury:**<br>▪ Damage to the growth plate around the knee can disrupt normal bone growth and lead to knock knees.<br>o **Obesity:**<br>▪ Excess weight can place increased stress on the knees, potentially contributing to knock knees.<br>o **Genetic Factors:**<br>▪ In some cases, genetic predispositions can play a role.<br><br>**Key Points to Remember:**<br><br>• Most knock knees in young children are a normal part of development.<br>• It's important to consult a doctor if:<br>o The knock knees are severe.<br>o They worsen after age 7.<br>o Only one leg is affected.<br>o The child experiences pain or difficulty walking.<br><br>I hope this information is helpful.	genetic conditions that affect bone growth and alignment.<br>3. **Rickets**: A bone growth problem caused by a deficiency in vitamin D, calcium, or phosphorus. This condition can lead to bone deformities, including knock knees.<br>4. **Infections**: Bone infections, such as osteomyelitis, can cause distortions in bone growth, leading to knock knees.

	• The child experiences **pain, difficulty walking, or frequent falls**.<br>• There is a noticeable difference between the two legs (**asymmetry**).<br><br>Would you like tips on exercises or treatments for knock knees?		5. **Injuries**: Trauma or fractures in the growth area of the bones around the knee can result in knock knees.<br>6. **Obesity**: Excess weight can put additional pressure on the knees, contributing to the development of knock knees.<br><br>If you have concerns about a child's knock knees, it's always a good idea to consult a healthcare professional for a proper diagnosis and guidance on any necessary treatments.<br><br>What should be done if a child has

			knock knees?Can you explain rickets and its effects on bone growth?How can knock knees be prevented in children?
13. How do I know if my child's knock knees are normal or need a doctor's help?	To determine whether your child's **knock knees** are normal or require medical attention, look for the following signs:<br><br>Signs That Knock Knees Are Normal:<br><br>✅ **Age 2-6:** Mild knock knees are common and usually correct themselves by age 7.<br>✅ **No pain or discomfort:** The child walks, runs, and plays normally without complaints.<br>✅ **Symmetrical alignment:** Both legs appear equally affected.<br>✅ **Improvement over time:** If the condition gradually gets better as the child grows.<br><br>When to See a Doctor:<br><br>❌ **Persists beyond age 7:** If the knock knees don't improve by this age.<br>❌ **Severe or worsening:** Knees touch significantly while feet are wide apart.	It's understandable to be concerned about your child's development. Here's a guide to help you determine when knock knees are normal and when it's time to seek a doctor's help:<br><br>**When Knock Knees Are Often Normal:**<br><br>• **Age Range:**<br>o Knock knees are common between ages 3 and 5.<br>o They often peak around age 4.<br>o They typically improve and resolve by age 7 or 8.<br>• **Symmetry:**<br>o Both legs are affected relatively equally.<br>o The child is otherwise developing normally.<br>• **No Pain or Limitations:**<br>o The child experiences no pain.<br>o They can walk, run, and play without significant difficulty.<br><br>**When to Seek a Doctor's Help:**	To determine if your child's knock knees are normal or if they need a doctor's evaluation, consider the following points:<br><br>Normal Knock Knees:<br><br>• **Age**: It's typical for children aged 2-7 to have knock knees as part of their natural growth process.<br>• **No Pain**: The child does not experience pain or discomfort while walking or running.

	❌ **Pain or walking difficulties:** The child complains of knee, hip, or ankle pain, has trouble walking, or frequently falls.<br>❌ **Uneven leg alignment:** If one leg is more affected than the other (**asymmetry**).<br>❌ **Family history or medical conditions:** If there is a family history of severe bone disorders or the child has had rickets, fractures, or joint diseases.<br><br>Next Steps If You're Concerned:<br><br>• A pediatrician or **orthopedic specialist** can assess the child's leg alignment.<br>• They may recommend **X-rays**, blood tests (for vitamin D levels), or gait analysis.<br>• Treatment options vary from **monitoring, exercises, braces**, or in rare cases, **surgery**.<br><br>Would you like suggestions on exercises to help with knock knees?	• **Age Concerns:**<br>○ Knock knees appear before age 2.<br>○ They persist or worsen after age 7.<br>• **Asymmetry:**<br>○ Only one leg is affected, or there's a significant difference between the two legs.<br>• **Pain or Functional Issues:**<br>○ The child experiences knee or hip pain.<br>○ They have difficulty walking or running.<br>○ They exhibit a limp.<br>• **Other Symptoms:**<br>○ The child is unusually short for their age.<br>○ There are signs of other underlying medical conditions.<br>• **Severe Knock Knees:**<br>○ If the distance between the ankles when the knees are together is very large.<br>• **Progressive Worsening:**<br>○ If you notice the condition is rapidly getting worse.<br><br>**Key Considerations:**<br><br>• A doctor can perform a physical examination and, if necessary, order X-rays to assess the severity of the knock knees and rule out any underlying medical conditions.	• **Symmetry**: Both legs show similar alignment without significant differences between them.<br>• **Improvement Over Time**: The condition appears to be improving as the child grows.<br><br>When to Seek Medical Advice:<br><br>• **Severe Deformity**: The knees are excessively angled inward or the condition seems more pronounced than usual.<br>• **Persistent Beyond Age 7**: The

		• Early diagnosis and intervention can help prevent potential complications.<br><br>In summary, while knock knees are often a normal part of childhood development, it's crucial to be aware of the signs that may indicate a more serious issue.<br><br>If you have any concerns, it's always best to consult with your child's pediatrician.<br>Sources and related content	knock knees persist beyond the age of 7 or show no signs of improvement.<br>• **Pain or Discomfort**: The child complains of knee pain, difficulty walking, or discomfort.<br>• **Asymmetry**: One leg is more affected than the other, indicating an uneven alignment.<br>• **Underlying Conditions**: Concerns about possible underlying conditions like rickets, infections, or trauma.

			What a Doctor Can Do:<br><br>• **Assessment**: A healthcare professional can perform a physical examination and review the child's medical history.<br>• **X-rays**: Imaging studies like X-rays can help determine the severity and underlying cause of the knock knees.<br>• **Treatment Plan**: If needed, the doctor can recommend treatments such as exercises, orthotics, or, in rare

			cases, surgery.<br><br>If you're ever in doubt or notice any of the concerning signs, it's always best to consult with a healthcare professional. They can provide peace of mind and ensure your child receives appropriate care.<br><br>What is normal knee alignment in children?What treatment options are available for knock knees?What other conditions can affect children's knees?
14. What tests do doctors use to check for knock knees?	If a doctor needs to evaluate your child's **knock knees**, they may use several tests to determine the cause and severity.<br><br>1. Physical Examination<br><br>◆ The doctor will check:<br><br>• Leg alignment while standing and walking.	When a doctor evaluates a child for knock knees, they typically use a combination of physical examination and, in some cases, imaging or laboratory tests. Here's a breakdown:<br><br>**1. Physical Examination:**<br><br>• **Visual Assessment:**	Doctors use several tests to diagnose knock knees and determine their severity. Here are some common methods:

	• Knee angle and spacing between the ankles.<br>• Any pain, stiffness, or difficulty moving.<br><br>2. Measurement Tests<br><br>📏 **Intermalleolar Distance (IMD):**<br><br>• The child stands with their knees touching.<br>• The doctor measures the distance between the ankles.<br>• A larger gap may indicate more severe knock knees.<br><br>3. Gait Analysis<br><br>👣 The doctor observes how the child walks to check for abnormal movement patterns or difficulty balancing.<br><br>4. X-rays or Imaging Tests *(if needed)*<br><br>📷 **X-ray (or MRI in rare cases):**<br><br>• Used if knock knees are severe, worsening, or only in one leg (**asymmetry**).	○ The doctor will observe the child's gait and how their legs align when standing.<br>○ They'll look for the characteristic inward angling of the knees.<br>• **Measurement:**<br>○ Doctors often measure the "intermalleolar distance," which is the distance between the ankles when the child stands with their knees together. This helps quantify the severity of the knock knees.<br>○ They will also observe leg length and general posture.<br>• **Assessment of Range of Motion:**<br>○ The doctor will check the range of motion in the child's knees and hips.<br><br>**2. Imaging Tests:**<br><br>• **X-rays:**<br>○ X-rays, particularly long-leg standing X-rays, can provide a clear picture of the bone alignment and help determine the degree of knock knees.<br><br>○ These images can also reveal any underlying bone abnormalities.	Physical Examination<br><br>• **Observation**: The doctor will observe the child's gait, posture, and leg alignment.<br>• **Measurement**: They may measure the distance between the ankles when the knees are together to assess the degree of knock knees.<br>• **Leg Strength and Flexibility**: Assessing the strength and flexibility of the leg muscles.

	• Helps detect bone deformities, growth plate issues, or past injuries.<br><br>5. Blood Tests *(if a nutritional or medical condition is suspected)*<br><br>🩸 **Vitamin D and Calcium Tests:** To check for **rickets**.<br>🩸 **Genetic or metabolic tests:** If a bone disease is suspected.<br><br>What Happens After Testing?<br><br>• If the knock knees are **mild and age-appropriate**, doctors usually recommend **observation and check-ups** over time.<br>• If there's an underlying issue (e.g., **rickets, Blount's disease, or injury**), treatment may include **bracing, physical therapy, or in rare cases, surgery**.<br><br>Would you like tips on home exercises or when to see a specialist? 😊	• In some instances, advanced imaging like CT or MRI scans may be used, though this is less common.<br><br>**3. Laboratory Tests:**<br><br>• **Blood Tests:**<br>○ If the doctor suspects an underlying medical condition, such as rickets (vitamin D deficiency), they may order blood tests to check vitamin D levels, calcium, and phosphorus.<br>○ Blood tests can also be used to evaluate for other metabolic bone diseases.<br><br>**Key Considerations:**<br><br>• In most cases, a physical examination is sufficient to diagnose knock knees.<br>• Imaging and laboratory tests are typically used when:<br>○ The knock knees are severe.<br>○ They are asymmetrical (affecting one leg more than the other).<br>○ There are concerns about an underlying medical condition.<br>○ The knock knees are outside of the normal age range for developmental knock knees.	Imaging Tests<br><br>• **X-rays**: X-rays are often used to get a detailed view of the bones and joints. This helps in identifying any underlying bone deformities or conditions.<br>• **MRI**: In some cases, an MRI may be used to get a more detailed image of the soft tissues and bones.<br><br>Additional Tests<br><br>• **Blood Tests**: To check for underlying conditions like rickets

		By using these methods, doctors can accurately assess knock knees and determine the appropriate course of action.<br><br>Sources and related content	or other metabolic disorders that might contribute to knock knees.<br><br>These tests help doctors determine the cause and severity of knock knees and guide the appropriate treatment plan2.<br><br>If you have any more questions or need further information, feel free to ask!
15. Will knock knees get better on their own as my child grows?	In most cases, **knock knees improve on their own** as a child grows. Here's what you need to know:<br><br>When Knock Knees Are Normal & Self-Correcting<br><br>✅ **Age 2-6:** Mild knock knees are common and part of normal development.<br>✅ **Age 7-8:** The legs usually straighten naturally without any treatment.	It's reassuring to know that in many cases, knock knees in children do indeed improve on their own as they grow. Here's a breakdown of what you can generally expect:<br><br>• **Normal Developmental Knock Knees:**<br>○ Most children experience knock knees as a natural part of their growth process, typically between the ages of 3 and 5.	In many cases, knock knees do improve on their own as children grow. Here's what you can generally expect:<br><br>Ages 2-7<br><br>During these early years, knock knees are often part of normal

	✅ **No pain or walking problems:** If your child is active and has no discomfort, it's likely a normal phase.<br><br>When Knock Knees May NOT Get Better on Their Own<br><br>❌ **Persists beyond age 7-8** without improvement.<br>❌ **Severe knock knees** (ankles far apart when standing straight).<br>❌ **Pain, difficulty walking, or frequent falls.**<br>❌ **Only one leg is affected (asymmetry).**<br>❌ **Family history of bone disorders or a past injury.**<br><br>What If They Don't Improve?<br><br>• If the condition doesn't correct by age **7-8**, see a doctor for evaluation.<br>• Treatments like **physical therapy, braces, or surgery (in rare cases)** may be considered for severe cases.<br><br>Would you like some **home exercises** that can help improve leg strength and alignment? 😊	◦ In the majority of these cases, the condition gradually corrects itself without any intervention.<br>◦ By the time a child reaches 7 or 8 years old, their legs usually straighten out to a normal alignment.<br>• **Factors Influencing Improvement:**<br>◦ The degree of knock knees: Milder cases are more likely to resolve on their own.<br>◦ The child's overall growth and development: Normal growth patterns often lead to natural correction.<br>• **When Medical Intervention Might Be Necessary:**<br>◦ If the knock knees are severe or worsen over time.<br>◦ If they persist beyond the typical age range.<br>◦ If they are accompanied by pain or other symptoms.<br>◦ If an underlying medical condition is identified.<br><br>Therefore, in many situations, parents can be reassured that their child's knock knees will improve naturally. However, it's always wise to consult with a pediatrician to ensure proper monitoring and to rule out any potential underlying issues.	development. Most children naturally outgrow this condition without any intervention.<br><br>Ages 7-10<br><br>By the age of 7 to 10, you should see a significant improvement in your child's leg alignment. If the knock knees are still present but are gradually correcting, this is typically a good sign.<br><br>Beyond Age 10<br><br>If the condition persists beyond the age of 10 or if it seems to be worsening, it's a good idea to consult a healthcare professional to ensure there are no underlying issues.

			Factors Influencing Improvement<br><br>• **Overall health**: A balanced diet rich in vitamins and minerals supports healthy bone development.<br>• **Physical activity**: Regular exercise can help strengthen the leg muscles and support proper alignment.<br><br>While many children do see improvement, it's always a good idea to keep an eye on their progress and consult a doctor if you have any concerns. They can provide reassurance

			and any necessary guidance.
16. What treatments are available for knock knees?	The treatment for **knock knees (genu valgum)** depends on the **child's age, severity**, and **underlying cause**. In most cases, knock knees correct themselves, but if they persist or cause problems, several treatments are available.<br><br>1. Observation & Monitoring 👀<br><br>• If knock knees are **mild and age-appropriate (2-6 years old)**, doctors usually recommend **waiting and watching**.<br>• Regular check-ups ensure the condition is improving naturally.<br><br>2. Exercises & Physical Therapy 🏃<br><br>• Strengthening leg muscles can **improve knee alignment**.<br>• Common exercises include:	The treatment approach for knock knees varies depending on the underlying cause, the severity of the condition, and the age of the individual. Here's an overview of the available treatments:<br><br>**1. Observation and Monitoring:**<br><br>• **For Normal Developmental Knock Knees:**<br>o In many cases, especially in young children between 3 and 5 years old, knock knees resolve naturally as they grow.<br>o Doctors often recommend regular checkups to monitor the progression and ensure it improves over time.<br><br>**2. Non-Surgical Treatments:**<br><br>• **Physical Therapy:**<br>o Exercises and stretches can help strengthen the muscles around the knees, improving stability and alignment.<br>o This can be beneficial for mild to moderate cases.<br>• **Orthotics:**<br>o Custom-made shoe inserts can provide additional	There are several treatment options available for knock knees, depending on the severity and underlying cause. Here are some common treatments:<br><br>Non-Surgical Treatments<br><br>1. **Observation**: In many cases, especially in young children, knock knees improve on their own as they grow. Regular monitoring by a healthcare professional can ensure proper development.<br>2. **Exercises and**

<table>
<tr>
<td rowspan="3"></td>
<td>✅ Side-Lying Leg Raises – Strengthens hip and thigh muscles.
✅ Squats & Lunges – Improve leg strength and balance.
✅ Bridging Exercises – Strengthens glutes and core.
✅ Stretching – Loosens tight inner thigh muscles.</td>
<td rowspan="3">support and improve foot and leg alignment.
Bracing:
In some cases, especially for children, braces may be used to guide the knees into a straighter position as they grow.
Treatment of Underlying Conditions:
If knock knees are caused by a condition like rickets, treatment will focus on addressing the underlying deficiency with vitamin D and calcium supplementation.
Weight management: If obesity is a contributing factor, weight loss can lessen the stress on the knees.
3. Surgical Treatments:
Guided Growth Surgery:
This minimally invasive procedure is used in growing children to slow down growth on one side of the growth plate, allowing the other side to catch up and straighten the leg.
Small metal plates or screws are used.
Osteotomy:
This surgical procedure involves cutting and</td>
<td rowspan="3">Physical Therapy: Specific exercises can help strengthen the muscles around the knees and improve leg alignment. Physical therapy may include stretches, strengthening exercises, and balance training.
3. Bracing: For children and adolescents with mild knock knees, wearing braces can help realign the knees and support proper growth.
4. Orthotics: Custom-</td>
</tr>
<tr>
<td>3. Bracing or Orthotics (for Growing Children) 🦿
Night braces: Used in some cases to guide bone growth.
Shoe inserts (orthotics): Help if foot positioning contributes to knock knees.
Usually recommended for children with severe knock knees or those with an underlying condition.</td>
</tr>
<tr>
<td></td>
</tr>
</table>

	4. Treating Underlying Causes<br><br>If knock knees are caused by an **underlying condition**, doctors may recommend:<br><br>• **Vitamin D & Calcium supplements** (for **rickets**).<br>• **Weight management** (if obesity is a factor).<br>• **Treatment for Blount's disease or arthritis** if diagnosed.<br><br>5. Surgery (Rare & Only for Severe Cases)<br><br>• **Guided growth surgery** – A small metal plate is placed on the knee to **slow growth on one side**, allowing natural correction.<br>• **Osteotomy** – In **adolescents or adults**, a bone is cut and realigned.<br>• Surgery is only considered if knock knees cause **pain, severe misalignment, or difficulty walking**.	realigning the bone to correct the angle of the leg.<br>○ It's typically used for severe cases or when knock knees persist after growth has stopped.<br>• Knee replacement: In older patients that also have arthritis in the knee, a knee replacement may be the best course of action.<br><br>**Important Considerations:**<br><br>• It's crucial to consult with an orthopedic specialist to determine the most appropriate treatment plan.<br>• Early diagnosis and intervention can help prevent potential complications.	made shoe inserts can help correct foot positioning and improve overall leg alignment.<br>5. **Weight Management**: Maintaining a healthy weight can reduce stress on the knees and improve alignment.<br><br>Surgical Treatments<br><br>1. **Guided Growth Surgery**: This minimally invasive procedure involves placing a small metal plate on the growth plate of the

	When to See a Doctor?<br><br>🚨 If your child has:<br>❌ Knock knees **beyond age 7-8** that don't improve.<br>❌ **Severe misalignment** (large gap between ankles).<br>❌ **Pain, difficulty walking, or frequent falls.**<br>❌ Only **one leg affected (asymmetry)**.<br><br>Would you like **detailed exercises** for strengthening the legs? 😊		knee to gradually correct the alignment as the child grows.<br>2. **Osteotomy**: In more severe cases, an osteotomy may be performed. This involves cutting and realigning the bones to correct the knee alignment.<br>3. **Total Knee Replacement**: In adults with severe knock knees and arthritis, a total knee replacement may be considered to relieve pain and improve function.

			Lifestyle Changes<br><br>1. **Healthy Diet**: Ensuring a diet rich in vitamins and minerals, especially vitamin D and calcium, supports bone health.<br>2. **Supportive Footwear**: Wearing shoes with good arch support can help improve leg alignment and reduce strain on the knees.<br><br>It's important to consult with a healthcare professional to determine the most appropriate treatment plan based on the individual's specific condition and needs. They can

			provide personalized recommendations and guidance.<br><br>If you have any more questions or need further information, feel free to ask!
17. What problems might happen if knock knees are not treated?	If **knock knees** (**genu valgum**) are mild and part of normal growth, they **usually don't cause problems** and correct themselves. However, if the condition is **severe, persistent, or left untreated** when necessary, it can lead to complications over time.<br><br>Possible Problems from Untreated Knock Knees<br><br>1. Knee & Joint Pain 🦵😖<br><br>• Uneven pressure on the knee joints can cause **pain, swelling, or stiffness**.<br>• May lead to **early arthritis** in the knees, hips, or ankles.	While many cases of knock knees resolve on their own, especially during childhood, untreated or severe cases can lead to various problems. Here's a breakdown of potential complications:<br><br>**1. Joint Problems:**<br><br>• **Osteoarthritis:**<br>○ The abnormal alignment of the knees can lead to uneven weight distribution, placing excessive stress on certain parts of the knee joint.<br>○ This can accelerate the wear and tear of the cartilage, increasing the risk of developing osteoarthritis, particularly in the knee.<br>• **Meniscus Tears:**<br>○ The altered knee alignment can also put extra strain on the menisci, the cartilage that cushions the knee.	If knock knees are not treated, several complications can arise, including:<br><br>1. **Joint Pain**: The misalignment can cause increased stress on the knees, leading to chronic pain and discomfort.<br>2. **Arthritis**: Over time, the abnormal pressure on the knee joints can lead to the development of

	2. Difficulty Walking or Running<br><br>• Severe knock knees can affect **balance and coordination**.<br>• Children may struggle with **running, jumping, or playing sports**.<br>• Increased risk of **frequent falls** or tripping.<br><br>3. Uneven Leg Wear & Posture Issues<br><br>• **Abnormal walking patterns** can cause **flat feet** or **overpronation** (feet rolling inward).<br>• Can lead to **back, hip, or foot pain** due to poor posture.<br>• Shoes may wear out **unevenly** due to the altered stance.<br><br>4. Long-Term Joint Damage (Osteoarthritis)<br><br>• Extra stress on the knee joint **wears down cartilage** over time.	o This increased stress can make them more susceptible to tears.<br>• **Patellar (Kneecap) Dislocation:**<br>o Knock knees can affect the tracking of the kneecap, increasing the risk of it dislocating.<br><br>**2. Pain and Discomfort:**<br><br>• **Knee Pain:**<br>o The misalignment can cause persistent knee pain, especially during or after physical activity.<br>• **Hip and Lower Back Pain:**<br>o The altered gait and posture caused by knock knees can also lead to pain in the hips and lower back.<br><br>**3. Gait and Mobility Issues:**<br><br>• **Gait Abnormalities:**<br>o Severe knock knees can affect a person's walking pattern, leading to an awkward or unstable gait.<br>• **Difficulty with Physical Activities:**<br>o The pain and instability associated with untreated knock knees can limit a person's ability to participate in physical activities.	osteoarthritis.<br>3. **Gait Issues**: Knock knees can affect the way a person walks, potentially leading to an abnormal gait and further musculoskeletal problems.<br>4. **Increased Risk of Injury**: The altered biomechanics can make the knees more susceptible to injuries such as ligament tears, meniscus tears, and cartilage damage.<br>5. **Reduced Mobility**:

	• May cause **osteoarthritis** at an earlier age, leading to chronic pain.<br><br>5. Psychological & Social Impact 😞<br><br>• Older children and teens may feel **self-conscious** about the way their legs look.<br>• Might **avoid sports** or social activities due to embarrassment or discomfort.<br><br>How to Prevent Complications? ✅<br><br>• **Regular check-ups** if knock knees persist past **age 7-8**.<br>• **Strengthening exercises** to support knee alignment.<br>• **Bracing or orthotics** if recommended by a doctor.<br>• **Early treatment for underlying conditions** (e.g., rickets, obesity).	**4. Other Potential Issues:**<br><br>• **Psychological Impact:**<br>o In some cases, the cosmetic appearance of knock knees can affect a person's self-esteem.<br>• in severe cases, and in rare instances, difficulty with walking.<br><br>**Key Points:**<br><br>• It's important to differentiate between normal developmental knock knees and those caused by underlying medical conditions.<br>• Early diagnosis and, if necessary, treatment can help prevent these potential complications.<br><br>Therefore, while many children outgrow knock knees, it's crucial to seek medical advice if you have any concerns.<br><br>Sources and related content	Severe knock knees can limit a person's ability to perform daily activities and reduce overall mobility.<br>6. **Psychological Impact**: The visible deformity and associated limitations can affect a person's self-esteem and mental well-being.<br><br>It's important to address knock knees early to prevent these potential complications and ensure proper leg alignment and function. If you have concerns, consulting a healthcare professional is the

	Would you like specific **exercises or home remedies** to help prevent problems? 😊		best course of action.<br><br>If you have any more questions or need further information, feel free to ask!
18. Do braces or other treatments work for knock knees?	es, **braces and other treatments** can help correct knock knees (**genu valgum**) in certain cases, especially in **growing children**. However, their effectiveness depends on the **child's age, severity of the condition, and underlying cause**.<br><br>1. Bracing for Knock Knees 🦵<br><br>✅ **When is it used?**<br><br>• Recommended for **children still growing** (usually under 10 years old).<br>• Helps guide the bones to **grow in a straighter alignment**.<br>• Often used in moderate-to-severe cases or if knock knees **are worsening**.	The effectiveness of braces and other treatments for knock knees depends heavily on several factors, including the child's age, the severity of the condition, and the underlying cause. Here's a breakdown:<br><br>**Bracing:**<br><br>• **Children:**<br>o Bracing can be effective in guiding bone growth in younger children whose bones are still developing.<br>o It aims to apply gentle pressure to encourage the legs to straighten over time.<br>o The success of bracing relies on consistent wear, as prescribed by the doctor.<br>• **Adults:**<br>o Bracing is generally less effective in adults, as their bones have finished growing.<br>o However, certain types of braces, such as "offloader" braces, can help relieve pain	Yes, braces and other treatments can be effective for knock knees, depending on the severity and underlying cause. Here's a breakdown of how these treatments work:<br><br>Braces<br><br>• **Purpose**: Braces help to realign the knees and support proper growth, especially in children and adolescents.<br>• **Effectiveness**: They can be

<table>
<tr>
<td rowspan="2"></td>
<td>✅ Types of Braces:

• Nighttime Orthotic Braces – Worn while sleeping to apply gentle pressure on the knees.
• Knee-Ankle-Foot Orthoses (KAFOs) – Used for more severe cases.

❌ Bracing is NOT effective for older children, teens, or adults because their bones have stopped growing.</td>
<td rowspan="2">associated with knock knees, especially when osteoarthritis is present. These braces shift weight away from the affected part of the knee.

Other Treatments:

• Physical Therapy:
o Exercises can strengthen the muscles surrounding the knees, improving stability and potentially aiding in alignment.
o This is beneficial for both children and adults, particularly in mild to moderate cases.
• Orthotics (Shoe Inserts):
o Orthotics can provide support and improve foot and leg alignment, which can indirectly help with knock knees.
o They can also help alleviate pain and improve gait.
• Weight Management:
o For individuals who are overweight or obese, losing weight can significantly reduce stress on the knees and improve symptoms.
• Surgical Intervention:
o In severe cases, or when conservative treatments fail, surgery may be necessary.</td>
<td rowspan="2">effective in mild to moderate cases of knock knees. The braces gently guide the legs into a straighter position over time.
• Usage: Typically worn for several hours a day, as recommended by a healthcare professional.

Other Non-Surgical Treatments

• Exercises and Physical Therapy: Strengthening the muscles around the knees can improve</td>
</tr>
<tr>
<td>2. Exercises & Physical Therapy

🔷 Helps strengthen leg muscles and improve knee alignment.
🔷 Common exercises:

• Side-lying leg raises – Strengthens hip muscles for better knee alignment.
• Squats & lunges – Build strength in the thighs and knees.
• Bridging exercises – Strengthens glutes and core for stability.</td>
</tr>
</table>

<table>
<tr>
<td rowspan="3"></td>
<td>Stretching – Helps loosen tight inner thigh muscles.
🔷 Works best for mild knock knees and preventing complications in more severe cases.</td>
<td rowspan="3">Surgical options include guided growth surgery for children and osteotomy for adults.
Surgical intervention is the most effective way to correct the bone alignment.
Key Considerations:
Early intervention is often more effective, especially in children.
A doctor's evaluation is essential to determine the most appropriate treatment plan.
Treatment effectiveness varies from person to person.
In summary, while braces and other treatments can be effective for knock knees, their success depends on individual circumstances. It's crucial to consult with an orthopedic specialist for proper diagnosis and personalized treatment recommendations.</td>
<td rowspan="3">alignment and support proper leg function. Exercises may include stretches, balance training, and specific strengthening routines.
Orthotics: Custom-made shoe inserts can correct foot positioning and improve overall leg alignment, reducing strain on the knees.
Weight Management: Maintaining a healthy weight can alleviate additional stress on the knees and</td>
</tr>
<tr>
<td>3. Orthotic Shoe Inserts (For Foot Support) 👟

✅ Used if knock knees are worsened by flat feet or poor foot alignment.
✅ Custom-made arch supports or shoe inserts help distribute weight properly.</td>
</tr>
<tr>
<td>4. Treating Underlying Causes 🏥
Rickets (Vitamin D Deficiency) → Vitamin D & calcium supplements.
Obesity-Related Knock Knees → Weight management & exercise.
Blount’s Disease or Severe Cases → May</td>
</tr>
</table>

	need **specialized treatment**.<br><br>5. Surgery (For Severe or Persistent Cases) 💉<br><br>• **Only for older children, teens, or adults** with **severe knock knees causing pain or mobility issues**.<br>• **Types of surgery:**<br>○ **Guided Growth Surgery** (small plates slow growth on one side of the knee).<br>○ **Osteotomy** (bone cutting and realignment for extreme cases).<br><br>Do These Treatments Work? 🤔<br><br>• **Mild cases** → Often improve naturally without treatment.<br>• **Bracing & orthotics** → Work best **in growing children** (under 10).		improve alignment.<br>• **Observation**: In many young children, knock knees improve naturally with growth. Regular monitoring by a healthcare professional can ensure proper development.<br><br>Surgical Treatments<br><br>• **Guided Growth Surgery**: This minimally invasive procedure uses small metal plates to correct knee alignment

	• **Exercises** → Help strengthen muscles & prevent complications.<br>• **Surgery** → Only needed if knock knees are severe and causing **pain or disability**.<br><br>Would you like **exercise recommendations** or help deciding if a doctor's visit is needed? 😊		as the child grows.<br>• **Osteotomy**: For severe cases, this surgery involves cutting and realigning the bones to correct the knee alignment.<br>• **Total Knee Replacement**: In adults with severe knock knees and arthritis, a total knee replacement may be considered to relieve pain and improve function.<br><br>The effectiveness of these treatments depends on the individual's specific condition. Consulting a healthcare professional is essential to

			determine the most appropriate treatment plan and achieve the best outcomes.<br><br>If you have any more questions or need further information, feel free to ask!
