# Supplementary material for: AI Chatbots in Pediatric Orthopedics: How Accurate Are Their Answers to Parents’ Questions on Bowlegs and Knock Knees?
Source: Healthcare (Basel). 2025 May 27;13(11):1271. doi: 10.3390/healthcare13111271 (PMC12154324; doi:10.3390/healthcare13111271)
Supplement: Supplementary file 1 [file healthcare-13-01271-s001.zip › Supplementory File S2.pdf]

Table (1) inter rater reliability assessment using intraclass correlation coefficient (ICC) for bowlegs responses

Domain	Chatpot	ICC (Avg)	95% CI (ICC)	p-value
**Accuracy**	**ChatGPT**	0.750	0.268–0.932	0.006
	**Gemeni**	0.825	0.487–0.953	<0.001
	**Copilot**	0.745	0.253–0.931	0.007
**Clarity**	**ChatGPT**	0.745	0.253–0.931	0.007
	**Gemeni**	0.646	−0.037–0.904	0.029
	**Copilot**	0.641	−0.050–0.903	0.031
**Comprehensiveness**	**ChatGPT**	0.812	0.449–0.949	0.001
	**Gemeni**	0.750	0.268–0.932	0.006
	**Copilot**	0.659	0.000–0.908	0.025
**Risk of Wrong answers**	**ChatGPT**	0.644	−0.042–0.904	0.030
	**Gemeni**	0.605	−0.158–0.893	0.045
	**Copilot**	0.594	−0.189–0.890	0.049

Table (2) inter rater reliability assessment using intraclass correlation coefficient (ICC) for Knock knees responses

Domain	Chatpot	ICC (Avg)	95% CI (ICC)	p-value
**Accuracy**	**ChatGPT**	0.594	−0.189–0.890	0.049
	**Gemeni**	0.793	0.395–0.944	0.002
	**Copilot**	0.605	−0.158–0.893	0.045
**Clarity**	**ChatGPT**	0.745	0.253–0.931	0.007
	**Gemeni**	0.646	−0.037–0.904	0.029
	**Copilot**	0.641	−0.050–0.903	0.031
**Comprehensiveness**	**ChatGPT**	0.671	0.036–0.911	0.021
	**Gemeni**	0.673	0.042–0.912	0.021
	**Copilot**	0.618	−0.118–0.897	0.039
**Risk of Wrong answers**	**ChatGPT**	0.644	−0.042–0.904	0.030
	**Gemeni**	0.600	−0.172–0.892	0.047
	**Copilot**	0.606	−0.156–0.893	0.044
